# Revealing the microbiome diversity and biocontrol potential of field *Aedes ssp*.: Implications for disease vector management

**DOI:** 10.1371/journal.pone.0302328

**Published:** 2024-04-29

**Authors:** Apolinar M. Hernández, Luis D. Alcaraz, Cristóbal Hernández-Álvarez, Miguel F. Romero, Angélica Jara-Servín, Hugo Barajas, Carlos M. . Ramírez, Mariana Peimbert

**Affiliations:** 1 Departamento de Ciencias Naturales, Universidad Autónoma Metropolitana, Unidad Cuajimalpa, Ciudad de México, México; 2 Posgrado en Ciencias Naturales e Ingeniería, Universidad Autónoma Metropolitana, Unidad Cuajimalpa, Ciudad de México, México; 3 Departamento de Biología Celular, Facultad de Ciencias, Universidad Nacional Autónoma de México, Ciudad de México, México; 4 Centro Regional de Control de Vectores, Panchimalco, Morelos, México; Uppsala University: Uppsala Universitet, SWEDEN

## Abstract

The mosquito *Aedes spp*. holds important relevance for human and animal health, as it serves as a vector for transmitting multiple diseases, including dengue and Zika virus. The microbiome’s impact on its host’s health and fitness is well known. However, most studies on mosquito microbiomes have been conducted in laboratory settings. We explored the mixed microbial communities within *Aedes spp*., utilizing the 16S rRNA gene for diversity analysis and shotgun metagenomics for functional genomics. Our samples, which included *Ae*. *aegypti* and *Ae*. *albopictus*, spanned various developmental stages—eggs, larvae, and adults—gathered from five semiurban areas in Mexico. Our findings revealed a substantial diversity of 8,346 operational taxonomic units (OTUs), representing 967 bacterial genera and 126,366 annotated proteins. The host developmental stage was identified as the primary factor associated with variations in the microbiome composition. Subsequently, we searched for genes and species involved in mosquito biocontrol. *Wolbachia* accounted for 9.6% of the 16S gene sequences. We observed a high diversity (203 OTUs) of *Wolbachia* strains commonly associated with mosquitoes, such as *wAlb*, with a noticeable increase in abundance during the adult stages. Notably, we detected the presence of the *cifA* and *cifB* genes, which are associated with *Wolbachia*’s cytoplasmic incompatibility, a biocontrol mechanism. Additionally, we identified 221 OTUs related to *Bacillus*, including strains linked to *B*. *thuringiensis*. Furthermore, we discovered multiple genes encoding insecticidal toxins, such as Cry, Mcf, Vip, and Vpp. Overall, our study contributes to the understanding of mosquito microbiome biodiversity and metabolic capabilities, which are essential for developing effective biocontrol strategies against this disease vector.

## Introduction

Mosquitoes are insects with complete metamorphosis (holometabolous) since their larvae differ remarkably from the adult state in their anatomy, ecology, and feeding. Mosquitoes have four life stages: eggs, larvae, and pupae that are aquatic and the adult stage that flies. The larvae feed on insects carcasses and debris [1], while adults have a proboscis that allows them to feed on nectars, and in some species, females feed on blood [2]. The site where mosquitoes develop has been reported as one of the factors that shape their microbiome [3–5]. However, the abundance of each bacterium within the microbiome is determined by the mosquito stage, sex, and species [6]. The microbiome of mosquito eggs comprises *Actinobacteria*, *Firmicutes*, and *Cyanobacteria* [7]. These phyla are also found in larvae; however, as the aquatic stages develop, the bacterial diversity increases [5]. The adults develop wings during metamorphosis, and the mouth structure changes completely. The change in the adult diet results in essential changes in the microbiome. In adults, the microbiome diversity decreases and is dominated by *Proteobacteria* [8]. The high carbohydrate concentration favors *Enterobacteria* growth [5]. In females, iron intake generates free radicals, increasing the abundance of *Enterobacteriaceae* and *Flavobacteriaceae* [5, 9, 10]. It is interesting that in the laboratory, *Ae*. *aegypti* adults from different parts of the world converge on a very similar microbiome [11]. In addition, vertical transmission of *Wolbachia* has been reported in mosquitoes. This intracellular bacterium has been identified in more than 60% of insects worldwide [12].

Recent studies on the gut microbiota of field collected *Aedes spp*. Mosquitoes reveal a limited diversity at higher taxonomic levels across various geographical regions. The free living adults are also dominated by *Proteobacteria* joined by *Actinobacteria*, *Bacteroidetes*, and *Firmicutes* [6]. Common bacterial families such as *Pseumonadaceae*, *Enterobacteriaceae*, *Acetobacteraceae*, *Rickettsiaceae*, and *Moraxellaceae* have been identified as widespread through these vectors [6, 13]. These findings are consistent across different species of wild mosquitoes, including *Anopheles spp*. [14]. Notably, inter-individual variability in microbiota composition is high, with some OTUs being exclusive to individual mosquitoes [13, 15]. Mosquito microbiota are afected by the blood source they feed on; human blood-fed mosquitoes harbor an array of human skin-associated bacteria [7]. It has been validated in the field that the microbiome of free-living larvae is determined by their habitat [16]. Interestingly, in the field, it has been observed that the microbial diversity of larvae has different taxa than that of adults before they feed [17]. Many of the studies in free living mosquito have focused on monitoring *Wolbachia* infection for control campaigns. *Wolbachia* genus is commonly found in *Ae*. *albopictus* [18]. However, in some countries of the world, the presence of *Wolbachia* in *Ae*. *aegypti* has not been detected [19], while in some studies, *Wolbachia* has been found [20, 21].

*Aedes spp*. is a global problem, as it transmits different diseases in humans and animals. They are reported as the main vector for dengue fever, chikungunya, and Zika virus [22]. Controlling mosquito populations is the most efficient way to prevent these viral diseases. Global climate change has allowed the displacement of mosquitoes to new places. *Aedes aegypti* that used to be in tropical regions are now detected in large cities in temperate regions [23]. The distribution of mosquitoes in these new places increases the need to improve the types of biocontrol strategies. Two bacterial biocontrol strategies have been successfully used to control mosquitoes: *Bacillus thuringiensis* as a larvicide [24] and *Wolbachia* spp. by interfering with the development of mosquitoes and protecting them from diseases [25]. *Bacillus thuringiensis* spores are added in food pellets to the water where the larvae grow [26]. Spore toxins kill larvae by forming pores in the midgut epithelium. These pores cause osmotic shock in the cells, leading to larval dehydration and death [27]. In contrast, *Wolbachia* gives rise to cytoplasmic incompatibility (CI). Embryo development is prevented when the male is infected and the female is not infected. *Wolbachia* has been used in two types of biocontrol strategies. In the first strategy, the incompatible insect technique (IIT), males transfected with a *Wolbachia* strain that does not exist in the wild are released; due to CI, these males reduce the mosquito population [28]. In the population replacement strategy (PRS), male and female mosquitoes are released with a Wolbachia strain that confers viral protection; this *Wolbachia* is fixed by CI [29].

Biocontrol strategies have been successful where they have been applied, but the different groups of microorganisms used as insecticides in mosquitoes have tended to lose effectiveness due to insect adaptation [26, 30]. Therefore, identifying new candidates for biocontrol use (whether new varieties of known species or new microorganisms) and determining the scope of the strategies already implemented in other insect populations is essential. Here, we describe the microbiome of *Aedes ssp*. in south-central Mexico and investigate the mosquito microbiome in real-world communities; our study remarks on the relevance of exploring microbial diversity in natural mosquito populations. Hence, we investigated bacterial diversity using 16S amplicons and microbial community metabolic capacity using shotgun metagenomics. Furthermore, we identified genes coding for insecticidal toxins carried by mosquitoes captured in semiurban environments. We defined the composition of bacteria previously reported as possible biocontrol agents. Our goal is to delve into overall diversity and coding genes to gain insights into mosquito-microbe interactions and contribute to the continuing development of microbe-directed mosquito biocontrol procedures.

## Materials and methods

### Sampling

We analyzed 14 samples, each representing a unique site and a specific developmental stage of mosquito populations. For each of the samples, we gathered 30 individuals to ensure enough DNA for metagenomic analysis. In the case of adult mosquitoes and larvae, all dissected organs were combined into a single sample. All larvae were obtained from the same water tank, and similarly, all eggs were collected from the same ovitrap. For the collection of adults, it was necessary to search for them in one to four houses within the same neighborhood.

Five localities in Mexico were sampled in June and July 2016 (rainy season). These localities have an incidence of dengue. The sampling sites were chosen based on the advice of the National Center for Disease Control and Prevention Programs (CENAPRECE). All the sites were semiurban single-family houses with roofs, ground-open water tanks, and electricity. The houses could have drainage, septic tanks, or latrines. Some houses had windows that were always open (no glass) or pets. Geographic location, temperature, and pressure data were collected (S1 Table in S1 File). Eggs were sampled from ovitraps that were one-liter plastic containers with a filter paper placed half way down. Females oviposited in the aerial paper section. Larvae were collected from the house’s water tanks or buckets, that were outdoors, using a plastic Pasteur pipette and deposited in jars with distilled water. Adult mosquitoes were collected from the indoors air with vacuum cleaners adapted with filters.

On the sampling day, the specimens were transported to the laboratory, adults and larvae were identified, and adults were separated by sex. The preliminary taxonomic analysis was conducted utilizing a stereoscopic microscope, coupled with the entomological expertise of CENAPRESE personnel. Larvae were classified by species employing dichotomous keys as per Mañez-Bernal & Martinez-Campos [31]. The species and sex of adults were determined using the pictorial key by Rueda [32]. While the intent was to exclusively collect *Ae*. *aegypti*, subsequent DNA analysis also revealed the presence of *Ae*. *albopictus*. The morphological assessment enabled us to exclude sites where *Culex* genus mosquitoes were prevalent.

During the four hours after the collection, the dissection was carried out. Larvae and adults were dissected to obtain intestines, salivary glands, ovaries, and Malpighian tubes [33]. Larvae were rinsed with water before disection procedure. Females displaying a red abdomen indicative of recent blood consumption were excluded to maximize the extraction of mosquito-derived bacterial DNA. No further analyses were conducted to determine whether the individuals had previously ingested blood. The organs of each individual were stored in batches of 30 individuals. The eggs were carefully removed with a clean spatula. Adult and larvae batches were resuspended in 3 ml of Hank’s salt medium (Sigma‒Aldrich) and kept at -80°C until processing. Eggs were sotored dry. The handling of the samples was carried out with gloves. All solutions, tubes, and tips were sterile, while work surfaces and laboratory equipment were constantly cleaned with 70% ethanol.

### Ethics statement

No specific permits were required to collect field mosquitos since *Ae*. *Aegipty*, *Ae albopictus* or *Culex* are not listed on the Red List of the International Union for Conservation of Nature (IUCN; http://www.iucnredlist.org/search). Moreover, mosquitoes, particularly *Aedes spp*. are classified within the national standard NOM-032-SSA2-2014 (for Epidemiological Surveillance, Promotion, Prevention And Control Of Vector Transmitted Diseases) as vectors requiring epidemiological surveillance and regular monitoring. The collection of eggs, larvae, and adult mosquitoes was carried out employing the methodology and as part of the control activities endorsed by the National Center for Disease Control and Prevention Programs (CENAPRECE), adhering to the guidelines set forth in the "Methodological Guide for Entomovirological Surveillance’’. We obtained oral consent from the homeowners where the sampling was conducted. The project has been registered within Universidad Autonoma Metropolitana under the number UAMC-DCN-47301018.

### DNA extraction and sequencing

DNA extraction was performed with PowerSoil DNA Isolation Kit (Qiagen) following the manufacturer’s instructions and optimized with phenol‒chloroform. Briefly, sample batches (approximately 250 μL) were vortexed for 10 min in bead tubes with 60 μL of C1 solution, 100 μL chloroform (Sigma‒Aldrich), and 100 μL phenol (Sigma‒Aldrich). Next, 16S ribosomal amplicons were generated using the 341F (5’-CCTACGGGNGGCWGCAG-3’) and 805R (5’-ACTACHVGGGTATCTAATCC-3’) primers. These amplified V3-V4 regions had a fragment size of approximately 464 bp. Amplicon sequencing was performed using the MiSeq Illumina platform (2x300 bp). Microbial DNA enrichment was performed for shotgun sequencing using the NEBNext® Microbiome DNA Enrichment Kit (New England Biolabs, NEB) following the manufacturer’s instructions. For shotgun sequencing, the Illumina NextSeq system (2x150) was utilized. Both 16S amplicon and shotgun sequences were generated using the LABSERGEN Langebio Cinvestav Irapuato platform.

### Sequence processing

Raw amplicon sequences were pair-end merged using CASPER [34]. Joined sequences were matched for 97% similarity using CD-HIT-EST [35]. One sequences for each OTU was selected using QIIME [36]. Taxonomic assignment was performed using the SILVA [37] database. Singletons and chimeras were removed using Chimera Slayer [38].

Shotgun reads were host filtered with bowtie2. Sequences were aligned with the genome from *Ae*. *aegypti* (strain LVP_AGWG, PRJNA318737). Subsequently, all nonhost sequences were quality filtered with Trimmomatic. The resulting sequences were assembled using SPADES [39]. A second assembly was performed with Velvet [40] for sequences discarded by SPADES. The resulting sequences (contigs and unassembled sequences) were annotated using BLAST [41] and DIAMOND [42] using the nonredundant M5nr database. The annotation obtained was compared with the application programming interfaces (APIs) of interest: PATRIC [43], RefSeq [44], and Ontology [45]. The taxonomic assignation was performed using Kraken [46]. The Bacterial Pesticidal Protein Resource Center database was used for toxin identification [47]. The molecular taxonomic classification of the mosquitoes was performed by identifying the ITS2 sequences directly from the reads, to avoid any chimera, followed by a BLAST against the RefSeq database [44]. Genome recruitments were performed from the raw sequences per sample using bowtie2 [48] and compared with reference genomes to determine resemblance and abundance within the metagenomes. The bacterial reference genomes used were *Wolbachia wMel* from *Drosophila melanogaster* ASM1658442v1 (NCBI NZ_CP046925.1), *Wolbachia wPip* from *Culex quinquefasciatus* (NCBI PRJNA30313), and *Wolbachia wAlbB* from *Ae*. *aegypti* (NCBI PRJEA76855).

### Statistical analysis

After the construction of the OTUs, ASV and shotgun metagenomic data were processed using R project standard functions or R packages such as "phyloseq2" [49] and "ggplot2" [50]. Alpha diversity metrics were calculated using the OTUs generated from the amplicon sequences of the 16S ribosomal gene, utilizing the “phyloseq2” package. Comparative analyses were performed, generating upset graphs and nonmetric dimensional scaling using Bray‒Curtis distance.

### Phylogenetic analysis

The OTUs assigned to *Wolbachia* and *Bacillus* were compared with the closest sequences of reference-type strains obtained from RDP [51] and RefSeq [44]. The sequences of the two OTU groups and their references were aligned using SSU-align [52], and the trees were built using FastTree [53] with default options (NJ and 1000 resamples). The trees were edited and annotated for presentation with iTol [54].

## Results

### *Aedes spp*. microbiome general description

All mosquitoes were collected from five locations in Mexico (S1 Fig in S1 File). These semiurban areas are characterized by a warm subhumid climate. Our study analyzed 14 samples, each representing a unique site and a specific developmental stage of the mosquito lifecycle, including eggs, larvae, female adults, and male adults. For each sample, we collected 30 individuals. Unfortunately, larvae and eggs were sourced from only two locations. Prior to molecular analysis, we had performed a taxonomic identification of the *Aedes spp*. Mosquitoes by morphological characterization. Of the 14 samples subjected to 16S gene amplicon analysis, eight underwent additional shotgun sequencing, encompassing all life stages of mosquitoes from the two sites. Through ITS2 analysis on these eight samples, we determined that four samples consisted exclusively of *Ae*. *aegypti* (samples: larvae_s1, larvae_s2, females_s1, male_s1*)*, while the other four were a mix of *Ae*. *albopictus* and *Ae*. *aegypti* (samples: eggs_s1, eggs_s2, females_s2, male_s2) (S1 Table in S1 File). We obtained 1,882,521 amplicon reads of the 16S gene clustered into 8,346 OTUs from 967 genera. In addition, we obtained 118,220,137 filtered reads from shotgun sequencing, from which 126,366 bacterial proteins were annotated (S2 Table in S1 File). We also analyzed the diversity using ASV to enable these data to be compared with many other datasets. The general descriptions are similar, although the precise numbers change. The most significant difference with the OTU analysis lies in the exclusion of rare organisms. We detected 4,829 ASVs from 563 genera (S2 Table in S1 File, and S2 Fig in S1 File).

**Fig 1:**
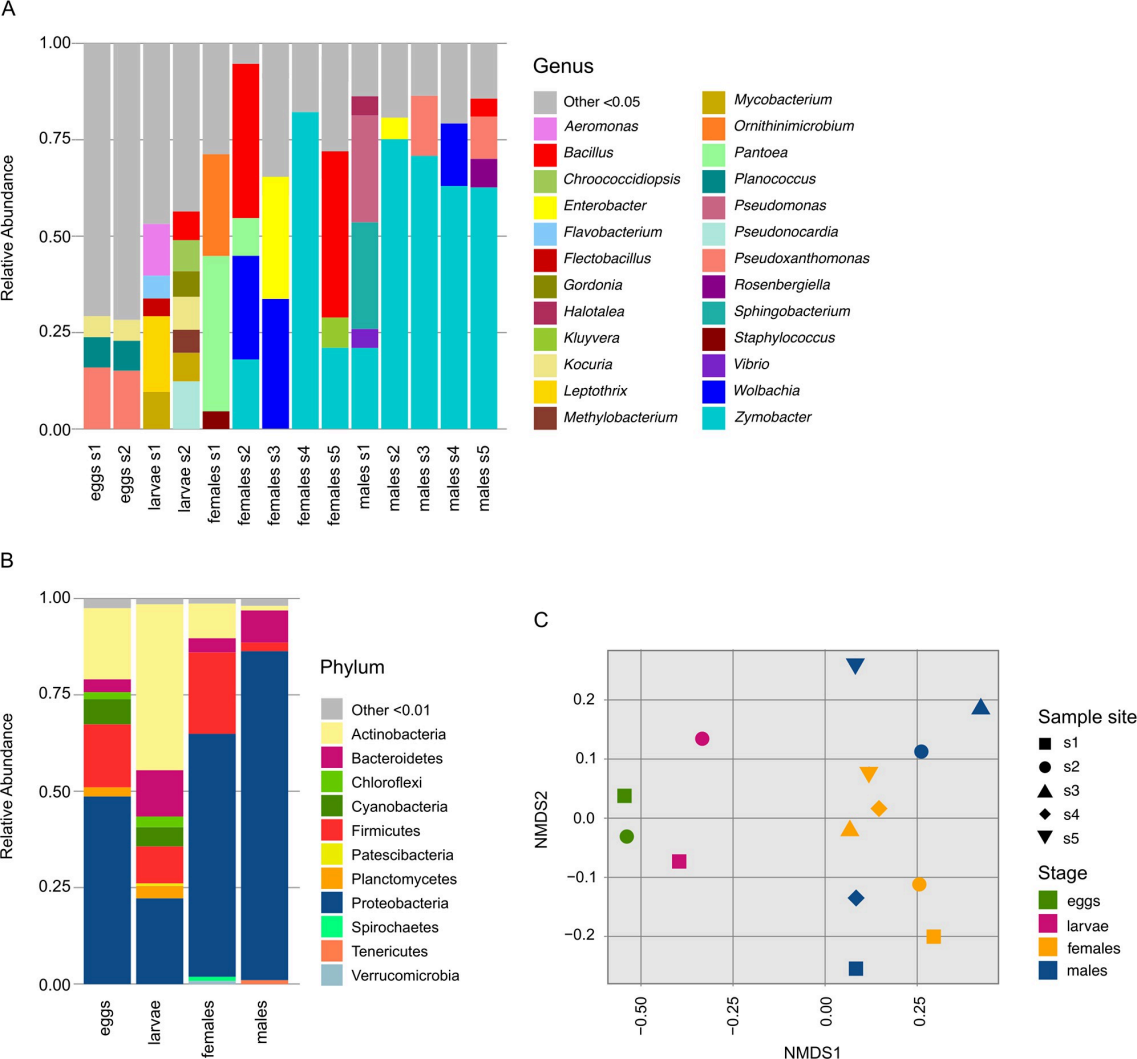
Phylogenetic profile of *Ae*. *aegypti* microbiome from 16S amplicon sequences. A) Genus distribution. B) Phyla distribution. C) Beta diversity plot (nonmetric multidimensional scaling, NMDS).

Alpha diversity indexes were estimated through analysis of the 16S data. Diversity during the aquatic life stages (Simpson index 4.7 ± 0.4) exceeded that observed in adult stages (Simpson index 2.0 ± 0.5). This pattern of diversity was consistent across all assessed metrics (richness, Shannon, Simpson, and Chao1) (S3, S4 Tables in S1 File). Amplicon analysis showed that the phylum *Proteobacteria* was the most abundant (67.8%), whereas the other phyla had much lower values, i.e., *Actinobacteria* (11.3%), *Firmicutes* (10.3%), *Bacteroidetes* (5%), and *Cyanobacteria* (1.3%). *Proteobacteria* were more abundant in the adult stages (73.3% adults and 35.8% aquatic), and *Actinobacteria* were more abundant in the aquatic stages (5.6% adults and 30.1% aquatic). Interestingly, photosynthetic *Cyanobacteria* and *Chloroflexi* were more abundant in the aquatic stages (Fig 1, S3 Fig in S1 File, and S5 Table in S1 File). Shotgun reads were also classified taxonomically.

The predominant OTUs in the complete system are those abundant in adult mosquitoes, whereas they are not the dominant ones in eggs and larvae. The dominant adult OTUs corresponded to the genera *Zymobacter sp*., *Bacillus sp*., *Wolbachia sp*., *Enterobacter sp*., and *Ornithimicrobium sp*., whereas *Pseudoxanthomonas sp*. was dominant in eggs (S6 Table in S1 File). The comparative set analysis shows that 190 genera were shared across developmental stages, although only 16 were detected in all samples. The eggs were the most diverse and had 223 unique genera. Despite being in the same developmental stage, only 288 genera were shared between females and males, whereas eggs and larvae shared 392 genera, including the 190 ubiquitous genera (S4 Fig in S1 File).

Metagenome analysis showed that 49.7% of the shotgun reads belonged to bacteria. We detected many fungal sequences, and 43.5% of the shotgun sequences belonged to the phylum *Ascomycota*. Bacteria were more abundant in the adult stages (70.5% adults and 28.9% aquatic) (S7 Table in S1 File). We grouped the samples by developmental stage and not by sample location because the beta diversity analyses suggested that there was community similarity (Fig 1, and S5 Fig in S1 File).

**Fig 2:**
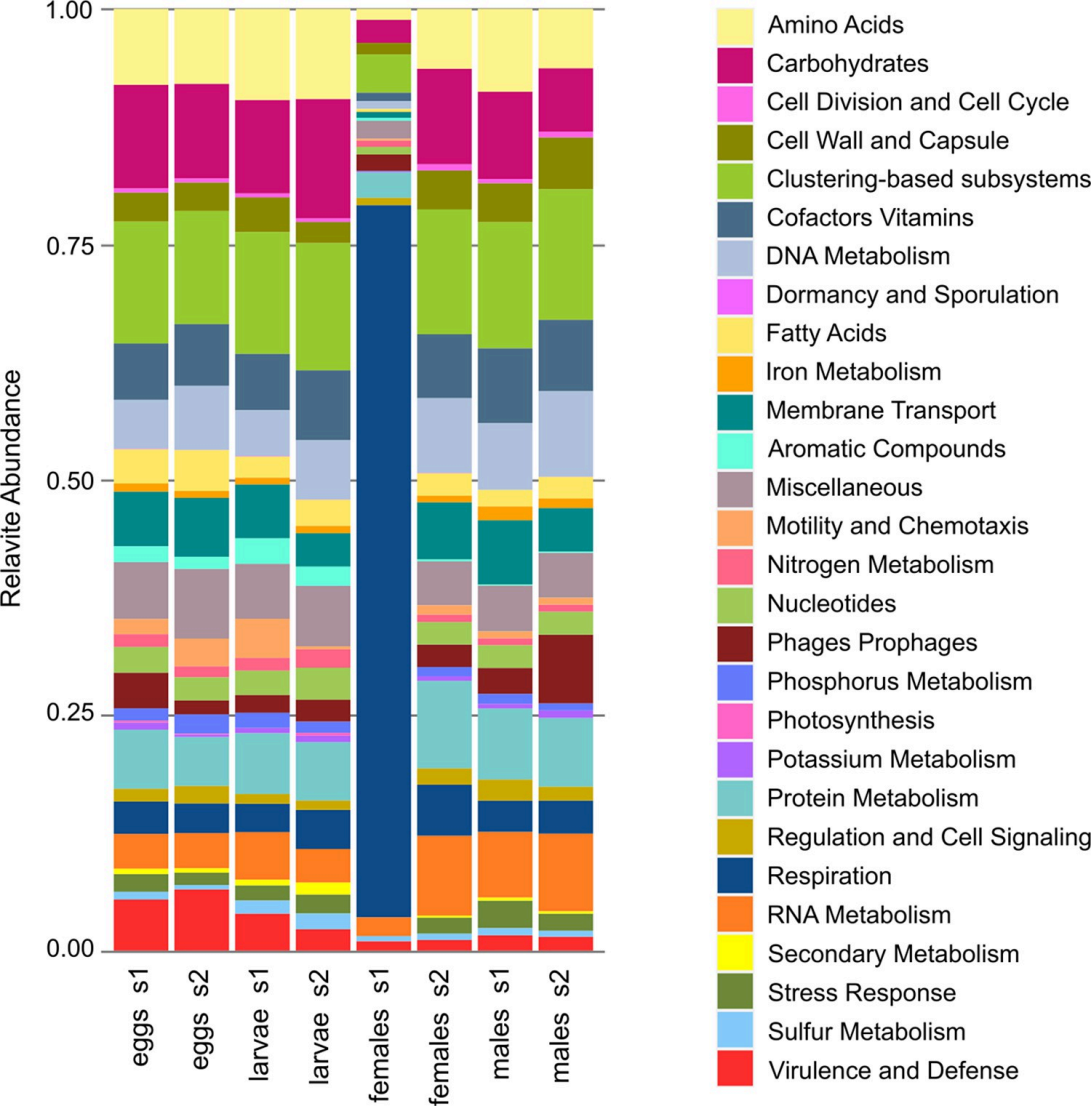
*Ae*. *aegypti* metabolic profiles from shotgun sequences. The SEED subsystems are shown to describe the main metabolic features.

Through the analysis of metagenomes, we assessed the metabolic capabilities of the microbiome. Notably, the ’female_s1’ sample exhibited a significantly higher proportion of genes associated with respiration, accounting for 76%, an evident contrast to the 3–5% observed in other samples (Fig 2). This rise in respiration is primarily attributed to an increase in the gene for cytochrome oxidases and respiratory complex I (S6 Fig in S1 File). Intriguingly, genes related to virulence and defense were found to be more prevalent during the egg and larvae stages, with percentages ranging from 3 to 6%, than in adults, where they accounted for only 0.9 to 1.6%. This suggests a reduction in these genes as development progresses. Within this metabolic category, we also noted a shift in gene types; eggs and larvae predominantly featured ton and tol trasport genes and efflux pumps, whereas in adults, type I and IV secretion systems were more abundant. In aquatic samples, phosphate metabolism genes were overrepresented, whereas adults showed a higher abundance of maltose and maltodextrin utilization genes. Certain genes, such as those for DNA replication and peptidoglycan synthesis, were highly represented across all samples. Across the entire system, the most prevalent metabolic category comprised gene groups (i.e. clustering based subsystems) that are not yet well-described, underscoring the importance of continuing research in bacterial physiology and classical genetics (Fig 2). Beta diversity analyses, including NMDS and PCoA of the metagenomic data, demonstrated that samples clustered according to the developmental stage (S7 Fig in S1 File).

### Potential biocontrol agents

We searched for microbes and genes that could be used for the biocontrol of mosquitoes. We incorporated insights from comprehensive reviews by Scolari *et al*., [6] and Gao *et al*., [55], which highlighted a spectrum of microbes already identified as pathogens [6, 55]. Additionally, we inspected the classifications of entomopathogenic activity mechanisms, drawing upon the frameworks set by Crickmore *et al*., [47] and the Bacterial Pesticidal Protein Database [47]. Our search was targeted towards microbes and genes that held potential for the biocontrol of mosquitoes, leading to the identification of multiple genera of interest due to their known insecticidal properties and relevance to biocontrol. We identified multiple bacteria with known biocontrol interest: *Bacillus*, *Wolbachia*, *Serratia*, *Enterobacter*, *Spiroplasma*, *Rickettsia*, *Lysinibacillus*, and *Clostridium*. We also identified several genera of fungi already reported to have entomopathogenic activity: *Smittium*, *Conidiobolus*, *Metarhizium*, *Tolypocladium*, *Pythium*, and *Beauveria*.

**Fig 3:**
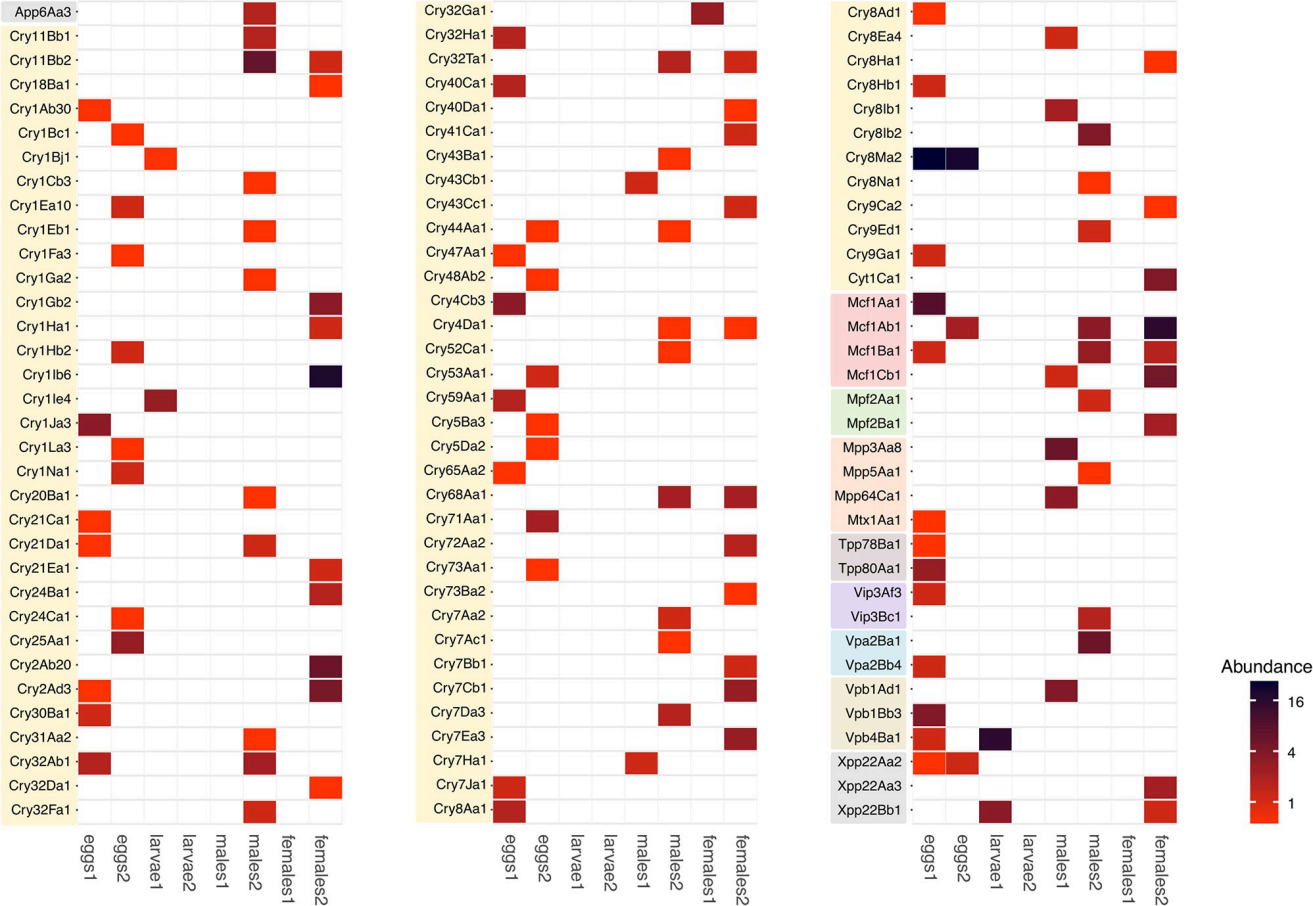
Insecticidal toxins detected in metagenomic shotgun sequences. The heatmap shows the observed sequences annotated as toxins in the Bacterial Pesticidal Protein Resource Center database. Each toxin type is highlighted in different colors. The Crystal type (Cry) protein family encompasses 78 representative sequences.

We identified 519 contigs containing coding genes for biosynthesis of 102 different toxins (Fig 3). The Crystal type (Cry) protein was the most abundant insecticidal toxin, with 371 sequences assigned to 78 Cry protein families. Cry8Ma2 stood out with 87 sequences, and Cry1Ib6 had 35 sequences. The other group of frequent toxins was the Make Caterpillars Floppy (Mcf) group, with 74 sequences assigned to four different Mcf clusters. Other groups of toxins identified included alpha-helical pesticidal protein (App); nonspecific cytolytic (Cyt); membrane attack complex/perforin (Mpf); Mtx2-related pesticidal protein (Mpp); mosquitocidal Mtx1 protein (Mtx); toxin-10 pesticidal proteins (Tpp); vegetative insecticidal protein (Vip); Vip2, the active component of the Vpa/Vpb binary pesticidal protein (Vpa); Vip1, the binding domain of the Vpa/Vpb binary pesticidal protein (Vpb) and holding class for pesticidal proteins (Xpp) (Fig 3). The samples with the most toxins were the eggs from site 2 (29 toxins, 129 sequences) and the females from site 2 (28 toxins, 157 sequences). In general, each toxin was observed only in one sample. Two toxin genes were shared between egg samples and four between females and males from site 2. In one larval sample, no toxin was detected, and in one female sample, only one gene was detected (Fig 3).

**Fig 4:**
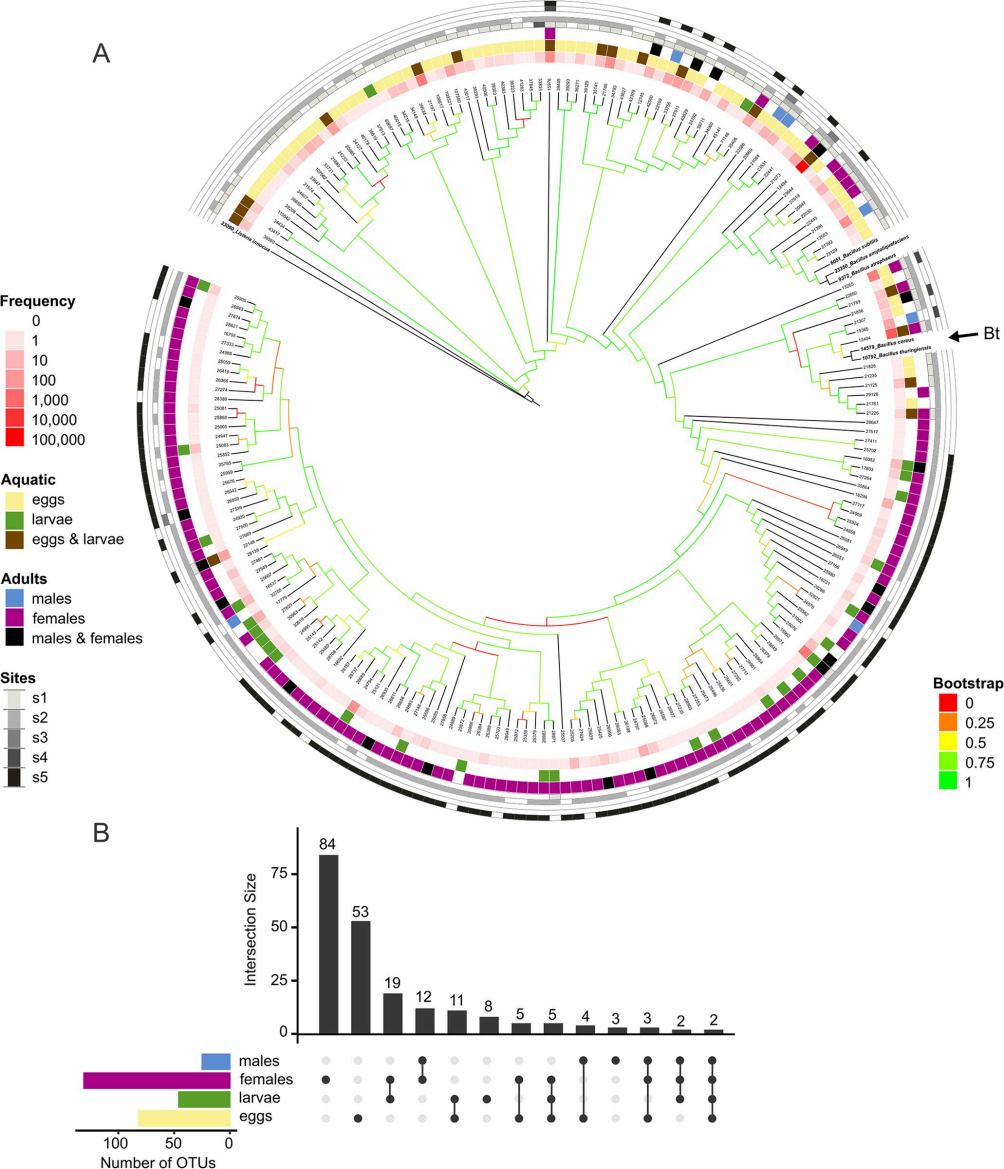
*Bacillus* diversity in mosquito microbiomes. A) Neighbor-joining tree of the Bacillus OTUs along with ATCC reference strains of *Bacillus thuringiensis*, *B*. *cereus*, *B*. *amyloliquefaciens*, *B*. *subtilis*, and *B*. *atrophaeus*. B) UpSet diagram of the distribution of Bacillus shared OTUs.

A dominant group in the mosquito microbiome was the genus *Bacillus*. We identified 221 OTUs from *Bacillus*, representing 12% of sequences (143,775) (S8 Table in S1 File). *Bacillus* OTUs were identified as *B*. *cereus*, *B*. *pumilus*, *B*. *firmus*, *B*. *aquimaris*, *B*. *vallismortis*, *B*. *thuringiensis*, and *Bacillus sp*. We constructed a 16S phylogenetic tree with the *Bacillus* sequences to describe their diversity in the mosquito microbiome. The *Bacillus* tree showed that the populations were divided into two groups: eggs and females. To identify the phylogenetic placement of the OTUs, the tree included sequences from reference strains. There were 13 Bacilli in the clade of *B*. *cereus* that came from aquatic and adult stages. However, none were identical to *B*. *cereus* or *B*. *thuringiensis* type strains. Only two OTUs were detected in all four stages, including the most abundant, corresponding to *B*. *seohaeanensis* and *B*. *pseudofirmus* (Fig 4).

**Fig 5:**
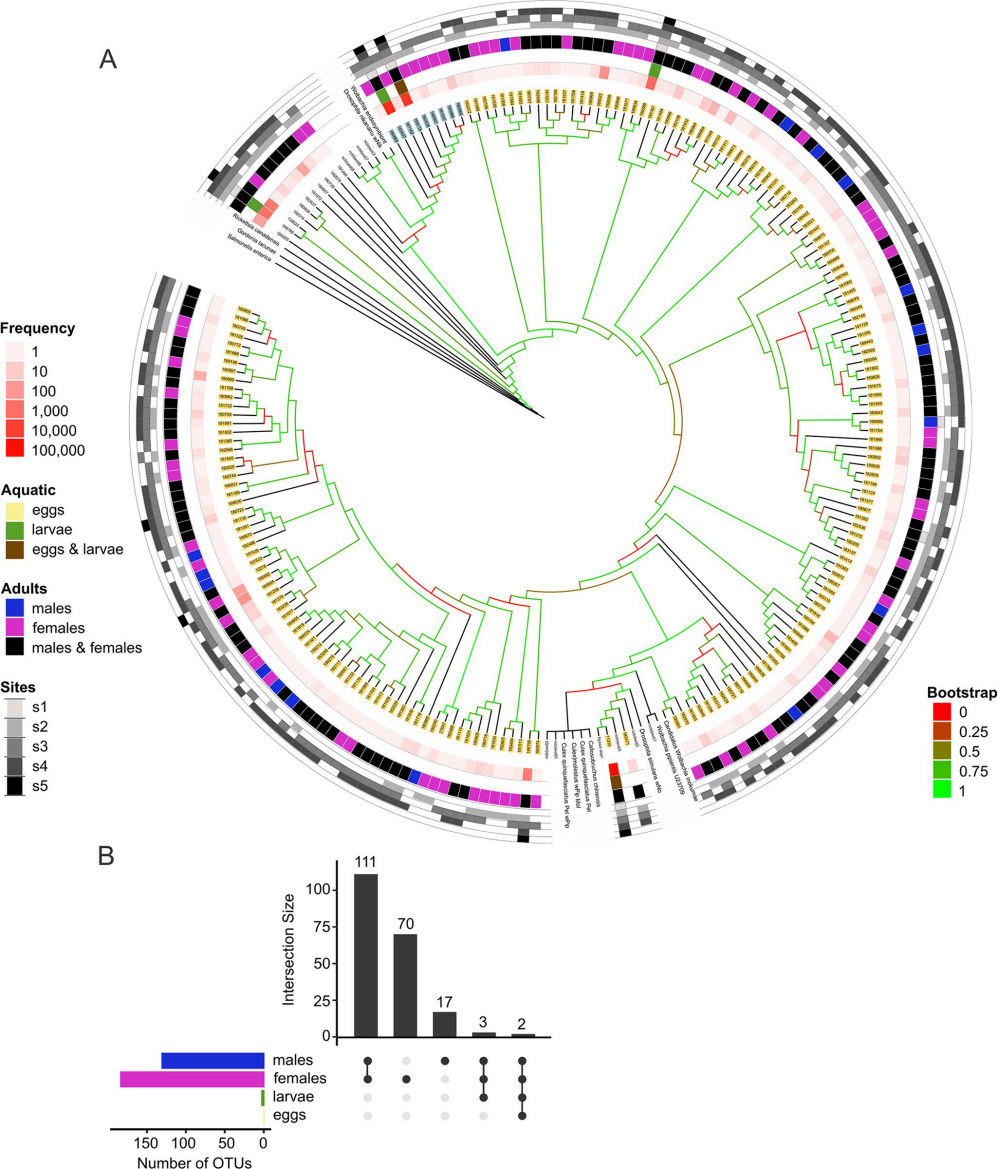
*Wolbachia* diversity in mosquito microbiomes. A) Neighbor-joining tree of the *Wolbachia* 16S rRNA gene OTUs. *Rickettsia canadensis*, *Gordonia lacunae*, and *Salmonella enterica* were used as outgroups. OTUs from Supergroup A are highlighted in blue, while Supergroup B is highlighted in yellow. B) UpSet diagram of the distribution of *Wolbachia* shared OTUs.

The genus *Wolbachia* was found in various samples in a nonhomogeneous pattern. We assigned 203 *Wolbachia* OTUs with more than two occurrences from 115,296 sequences (S9 Table in S1 File). These constituted 9.6% of the total system. However, the *Wolbachia* abundance in each sample ranged from 0 to 32%. In the sample ’males_s2,’ no OTUs were detected despite the identification of *Ae*. *albopictus* within that sample. While in the samples exclusive to *Ae*. *aegypti*, only six *Wolbachia* OTUs were present, which also appeared in other samples. Adult samples exhibited a shared presence of 116 OTUs. As expected, the presence of *Wolbachia* in aquatic stages was minimal for all samples (0.037%). *Wolbachia* was not found in males from site 2. In contrast, females from site 3 showed 158 *Wolbachia* OTUs. *Wolbachia* abundances or diversities were not similar within a sampling location or sex (S9 Table in S1 File). A phylogenetic tree was constructed for a more detailed description, including reference sequences (Fig 5). The tree showed that the OTUs corresponded primarily to strains of *Wolbachia* from supergroup B, and 9 OTUs were associated with supergroup A. However, both supergroups were abundant and prevalent among *Wolbachia* OTUs.

**Fig 6:**
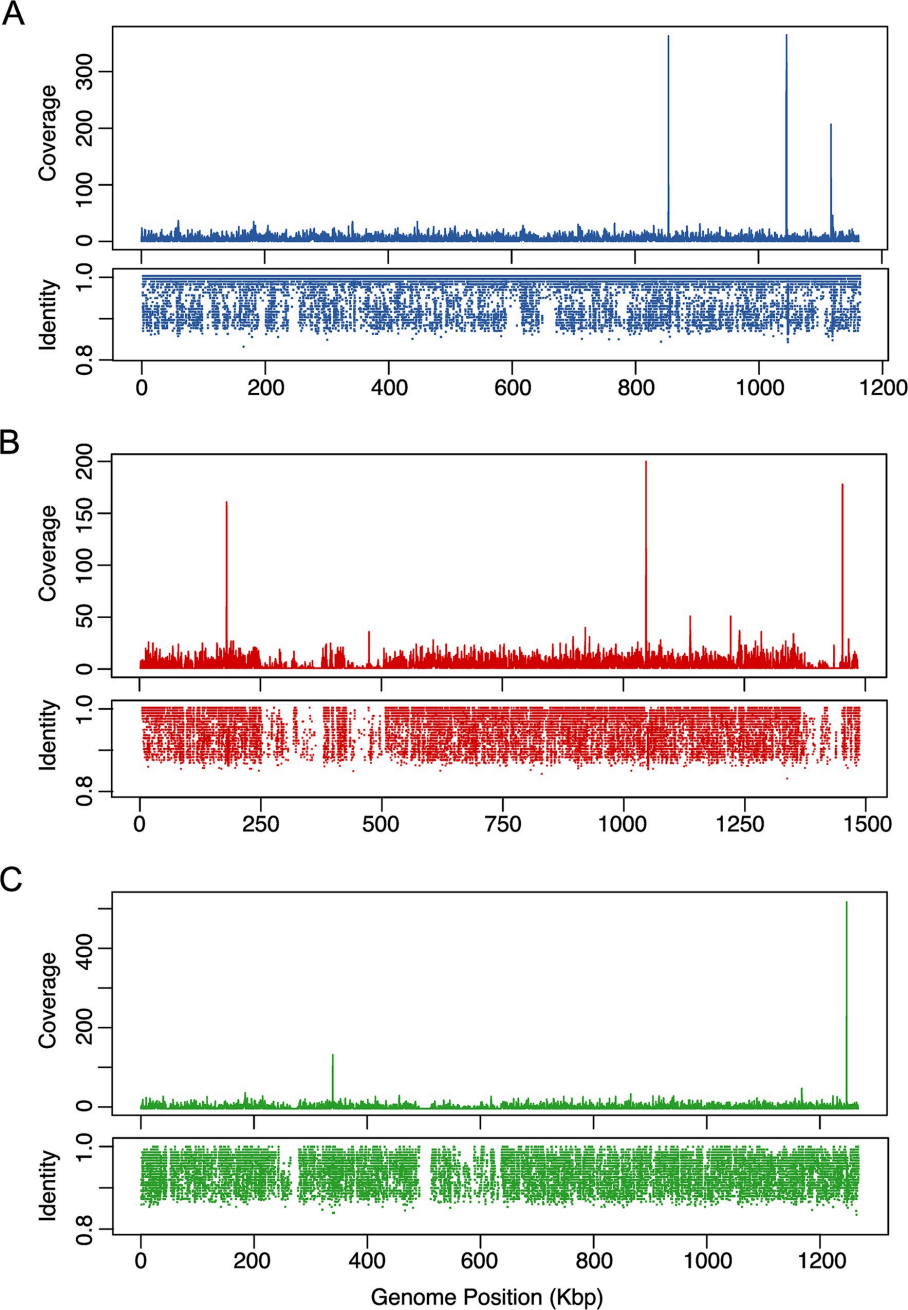
Recruitment of *Wolbachia*. *Wolbachia* genomes analyzed against the sample of females from site 2. A) *wAlbB* PRJEA76855. B) *wPip* PRJNA30313. C) *wMel* NZ_CP046925.1.

The recruitment of *Wolbachia* sequences in the metagenomes was conducted to understand similarities to *Wolbachia* previously isolated from *Aedes* mosquitoes and other Diptera, such as *Culex* and *Drosophila*. We used three strains of *Wolbachia* as reference genomes: *wAlbB* (PRJEA76855), *wPip* (PRJNA30313), and *wMel* (NZ_CP046925.1). The sample from site 2 presented more *Wolbachia* sequences, so the recruitment of that sample had better coverage. The genome coverage of the *wAlbB* strain was almost complete in the sample from the female from site 2 (*wAlbB* 83.05%, *wMel* 60.72%, and *wPip* 58.46%). The *wAlbB* recruitment showed a distribution across the entire genome of sequences with identities of 100%, so it is feasible that this strain was in this sample. Recruitment also confirmed *Wolbachia* diversity because there were many hits with identities between 85 and 99% throughout the genome (Fig 6 and S9 Table in S1 File).

We searched for cif genes associated with cytoplasmic incompatibility generated by *Wolbachia*. Cif genes were only identified in the adult samples from site 2, which were the samples with the highest number of *Wolbachia* sequences. We identified 425 sequences corresponding to four *cifA* and 16 types of *cifB*. The genes were similar to *Wolbachia* cif genes found in *Culex pipientis*, *Nasonia oneida*, and *Ceratitis capitata* (S9 Table in S1 File).

## Discussion

The microbiome of the *Aedes spp*. collected in Mexico was similar to those already reported for field and laboratory mosquitoes. *Proteobacteria*, *Actinobacteria*, and *Firmicutes* were the most abundant phyla; together, they accounted for 89% of the system, and an increase in *Proteobacteria* was observed in the adult stage [6, 56–58]. We observed a doubling of *Proteobacteria* in adults (from 35 to 73%), which is expected due to the change in diet. For females, blood intake promotes the growth of bacteria tolerant to reducing oxide environments [9]. Genera exhibited a unique distribution pattern across each sample, with only 16 genera consistently present throughout. We observed a small core and a high proportion of rare OTUs, suggesting stochasticity within their natural environment [59]. This could also be indicative of intense competition among the bacteria or temporal instability within the bacterial communities. Studies focusing on isolated individuals have demonstrated significant variability between individuals, aligning with the observed small core [15, 60]. As in other studies, we detected that the most abundant egg genus is *Pseudodoxanthomonas* [56, 61]. *Mycobacterium* is the most abundant genus in larvae (in agreement with Zouache [16]). It is not typical for *Zymobacter* to be dominant in adults; however, its presence is typical [62], and in a field study in Malaysia, *Zymobacter* was also one of the most frequent bacteria [63].

Our beta diversity analyses demonstrated that the mosquito developmental stage is the primary determinant of the mosquito microbiome. However, other studies of field mosquitoes have suggested that the sampling site is a significant factor in shaping mosquito microbiomes [6]. In our case, the impact of the developmental stage appears to have been more substantial than that of the location or microenvironment characteristics. Additionally, the variability in species presence—some samples containing only one species and others two—also proved to be a less critical factor than the developmental stage. Yet, we cannot completely dismiss the potential influence of the collection site in this study, as only one sample per site per state was examined.

The female samples exhibited little resemblance to each other. In particular, the female sample S1 is dominated by *Pantoea* and *Ornithinimicrobium*. *Pantoea* is an aerobic bacterium; it is commonly found in insects, and has been reported to boost insect fitness by metabolizing a wide range of compounds, including toxic substances [64]. This ability to compete and survive in various environments makes numerous species within this genus attractive candidates for biocontrol and bioremediation applications [64]. *Ornithinimicrobium* is also aerobic, and remains largely uncharacterized in terms of its metabolic or ecological traits. The observed taxonomic diversity between female samples corresponds to distinct metabolic gene patterns. It is documented that adults’ microbiomes resemble those of pupae upon emergence; however, the microbiome undergoes a complete transformation after the first feeding and can be shaped by different blood meal sources or the elapsed time post-blood ingestion [5, 65, 66]. The microbiome of adult mosquitoes is molded by the accessibility of varied food sources, such as differences in nectar and blood [10]. Our sampling protocol does not provide the means to ascertain whether the females had previously fed or if other environmental factors may have influenced the microbiome. The substantial proportion of genes related to respiration in the female sample from site 1 suggests that these mosquitoes have likely blood fed. Aquatic samples display a more diverse metabolism, and despite differences in bacterial genera, they show high metabolic resemblance. Genes associated with virulence, disease, and defense are found to be less prevalent in the adult stages.

The shotgun sequencing analysis also allowed us to detect fungi associated with these mosquitoes. In eggs and larvae, *Mycosphaerella* was widely dominant. *Mycosphaerella* is a pest found in *Diptera* from plant galls [67]. In adults, the most common fungi were *Aspergillus* and *Metarhizium*. *Aspergillus* is a ubiquitous filamentous fungus that is typically found in the soil but is also capable of colonizing insects such as *Tenebrio molitor*, *Apis mellifera*, and *Anopheles coluzzii* [68]. In *Ae*. *aegypti*, the *Aspergillus* prevalence was between 4 and 7% of adult samples. Interestingly, some species of the *Aspergillus* genus have been reported to have entomopathogenic functions in mosquitoes [69]. Like *Aspergillus*, *Metarhizium* is a soil fungus that includes entomopathogenic species. *M*. *anisopliae* is commercially produced as a biocontrol agent against agricultural pests, including mosquitoes [70]. In addition, *Metarhizium* coinfection decreased the load of dengue virus in female *Ae*. *aegypti* [71]. Moreover, transgenic *M*. *anisopliae* have been developed that prevent *Plasmodium* transmission [72]. The disadvantage of using *M*. *anisopliae* as a biocontrol agent is that it is not specific to mosquitoes [70]. It is worth mentioning that in our samples, 3.91 and 0.66% of the adult female sequences corresponded to *Plasmodium*.

Detection of *Wolbachia* in mosquitoes is highly variable. *Wolbachia* has been reported as a prevalent and the most abundant taxon for *Ae*. *albopictus* [8]. Numerous investigations have failed to identify *Wolbachia* in *Ae*. *Aegypti* [30, 73, 74]. Nevertheless, *Wolbachia* has been found in *Ae*. *aegypti* in the Philippines, Thailand, and Panamá in low abundance [21,59, 75]. Numerous studies have been directed towards detecting *Wolbachia*, given its significance for biocontrol. Most research has employed endpoint PCR using specific primers for *Wolbachia* supergroups A and B. Additionally, a variety of other methods have been utilized, including PCR, real-time PCR (qPCR), restriction fragment length polymorphism (RFLP), multilocus sequence typing (MLST), and massive sequencing of the 16S ribosomal gene [76]. A major limitation of amplifying specific sequences is the potential to miss other strains. For massive 16S sequence analyses, a notable constraint is that when using Amplicon Sequence Variants (ASVs), the less abundant sequences are often discarded [77]. These methodological differences could be the reason for the high *Wolbachia* diversity observed in our study.

Although we determined that the presence of *Wolbachia* was null for some samples and sites, in other samples, *Wolbachia* was one of the most abundant OTUs. We identified 203 OTUs, which constituted 6% of the amplicon sequences; most corresponded to the *Wolbachia* B supergroup, and nine OTUs corresponded to the A supergroup. *Wolbachia* strains are divided by molecular differences into eight phylogenetic supergroups (A-H). Wolbachia supergroups A and B are commonly associated with arthropods and have both been detected in *Aedes spp*. [78]; co-infection of both groups is even prevalent in *Ae*. *albopictus* [21]. Experiments using qPCR have demonstrated that group B is more abundant [66]. Genomic analyses indicate a low frequency of intergroup recombination, suggesting that the supergroups occupy distinct niches (Wang et al., 2020). The variation in strain diversity and the sporadic occurrence of Wolbachia imply that, at least for the less common strains, vertical transmission of *Wolbachia* in *Aedes ssp*. is inefficient and no solid symbiotic relationship promotes the natural selection of infected mosquitoes. In addition, only two strains were observed in eggs and five in larvae, suggesting that most *Wolbachia* colonization occurs in the adult stage.

Cytoplasmic incompatibility (CI) biocontrol in *Ae*. *aegypti* has been carried out with *Wolbachia*-free mosquitoes [79], but in mosquitoes naturally infected with *Wolbachia*, like *Ae*. *albopictus*, cytoplasmic incompatibility processes have been generated by crossing a different phylogenetic group from the same mosquito species or other species, such as *D*. *melanogaster* [80]. Notably, we did not observe the *wMel* strain used in biocontrol experiments in the field. In this work, we found that the diversity of sequences associated with *cifA* was much lower than that of *cifB* (4 and 16 genes, respectively). However, we do not know the potential activity of these genes to recover CI.

The *Wolbachia* distribution in *Aedes spp*. suggests that biocontrol by release from *Wolbachia*-transfected mosquitoes could have different efficiencies in different populations of *Aedes spp*. These biocontrol strategies depend on cytoplasmic incompatibility (CI), directly in the case of the incompatible insect technique (IIT) or to amplify the effect of the population replacement strategy (PRS). When vertical transfer is not efficient, CI is not efficient; the possibility of a diversity of *Wolbachia* in females could include rescue by *cifA* expression in females [81]. Cytoplasmic incompatibility rescue becomes less likely than vertical transmission when the introduced strain has a different phylogenetic origin, whereas vertical transmission efficiency can be improved with temperature-resistant *Wolbachia* strains [30]. Regardless, the results using PRS are impressive. In Indonesia, it was possible to reduce the incidence of dengue by 77% owing to the release of mosquitoes transfected with *wMel* [82].

We identified 221 *Bacillus* OTUs, of which 13 corresponded to the *Bacillus thuringiensis* and *B*. *cereus* groups. Because we detected 78 Cry toxins, we assumed the presence of Bt. We found that the *B*. *thuringiensis* clade is only abundant in the aquatic stages of the mosquito life cycle, which could indicate its adult toxicity. The genes for Cry and Mcf toxins found in different samples in apparently healthy specimens would not prevent consideration of these toxins or bacteria as biocontrol agents. In total, 102 toxins were identified, including Cry toxins. The gene sequences of these toxins differ from those previously reported, so samples from free-living mosquitos are an excellent source of toxin biodiversity.

We also detected other bacteria with mosquito biocontrol potential. *Spiroplasma* is a male-killing bacterium for *Coleoptera* [83]; *Serratia* prevents malaria in the mosquito and is an insect pathogen [84]; *Clostridium* restricts systemic CHIKV infection [85] and produces larvicidal toxins [86]; and *Lysinibacillus* can produce antimalarial siderophores [87]. We also found *Enterobacter*, which has been used to express transgenic toxins in the midgut mosquito (paratransgenic control) [88].

## Conclusion

We identified a source of microorganisms and toxins with insecticidal potential in different mosquito populations, mainly in eggs. We determined that the microbiome is strongly associated with the mosquito developmental stage. Additionally, we detected *Wolbachia* with the potential to generate and recover cytoplasmic incompatibility. Understanding the microbiome of wild mosquito eggs and larvae could be relevant for developing future biocontrol strategies.

## Supporting information

S1 File Supplementary figures and tables. (PDF)
