# Supplementary material for: Revealing the microbiome diversity and biocontrol potential of field Aedes ssp.: Implications for disease vector management
Source: PLoS One. 2024 Apr 29;19(4):e0302328. doi: 10.1371/journal.pone.0302328 (PMC11057774; doi:10.1371/journal.pone.0302328)
Supplement: S1 File — (PDF) [file pone.0302328.s001.pdf]

Supporting/Supplemental Information

Revealing the Microbiome Diversity and Biocontrol Potential of Field *Aedes ssp.*: Implications for Disease Vector Management

Apolinar M. Hernández[1, 2], Luis D. Alcaraz3, Cristóbal Hernández-Álvarez[3], Miguel F. Romero[3], Angélica Jara-Servín[3], Hugo Barajas[3], Carlos M. Ramírez[4], Mariana Peimbert[1*].

* corresponding author mpeimbert@cua.uam.mx

Supplementary material

Fig S1 Sample location maps.
Fig S2 ASVs diversity.
Fig S3 Phylogenetic profile (shotgun sequencing).
Fig S4 Beta diversity plots for OTUs.
Fig S5 OTUs Upset plot.
Fig S6 Metabolic pathways abundances from shotgun sequences.
Fig S7 Beta diversity plots for shotgun sequences.

Table S1. Samples metadata
Table S2. Sequencing effort and reads assignment summary.
Table S3. Alpha diversity indexes for OTUs.
Table S4. Alpha diversity indexes for ASVs.
Table S5. Phyla abundance from OTUs.
Table S6. Genera abundance from 16S sequences
Table S7. Phyla abundance from metagenome shotgun sequences.
Table S8. Abundance of Bacillus OTUs.
Table S9. Wolbachia sequences summary.

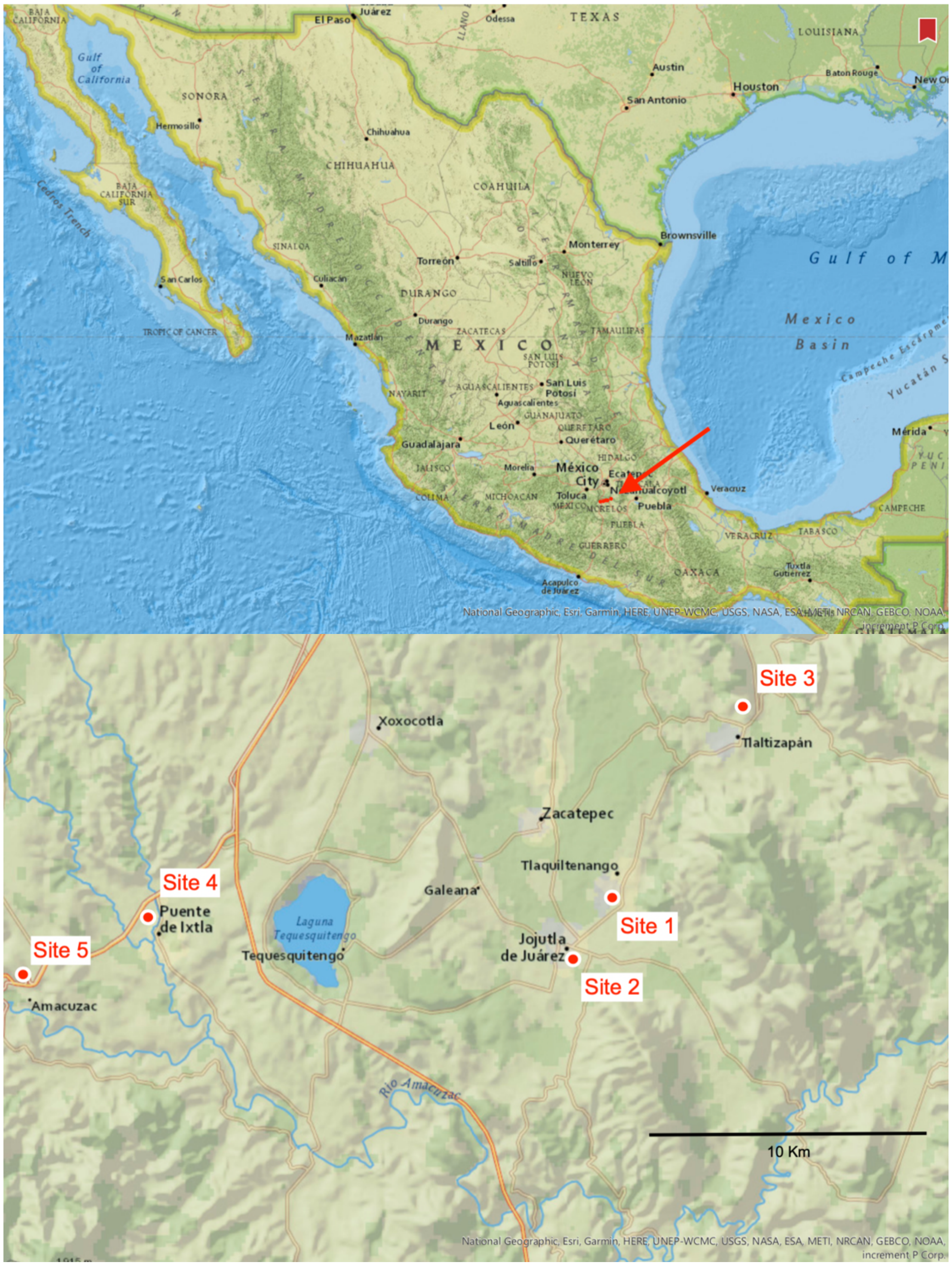

Fig. S1 Sample location maps. A. Political map. B. Satelital map (Site 1: 18.6236 N 99.1578 W, Site 2: 18.6089 N 99.1836 W, Site 3: 18.6865 N 99.1177 W, Site 4: 18.6247 N 99.3239 W, Site 5: 18.6030 N 99.3819 W) (https://www.usgs.gov/programs/national-geospatial-program/national-map)

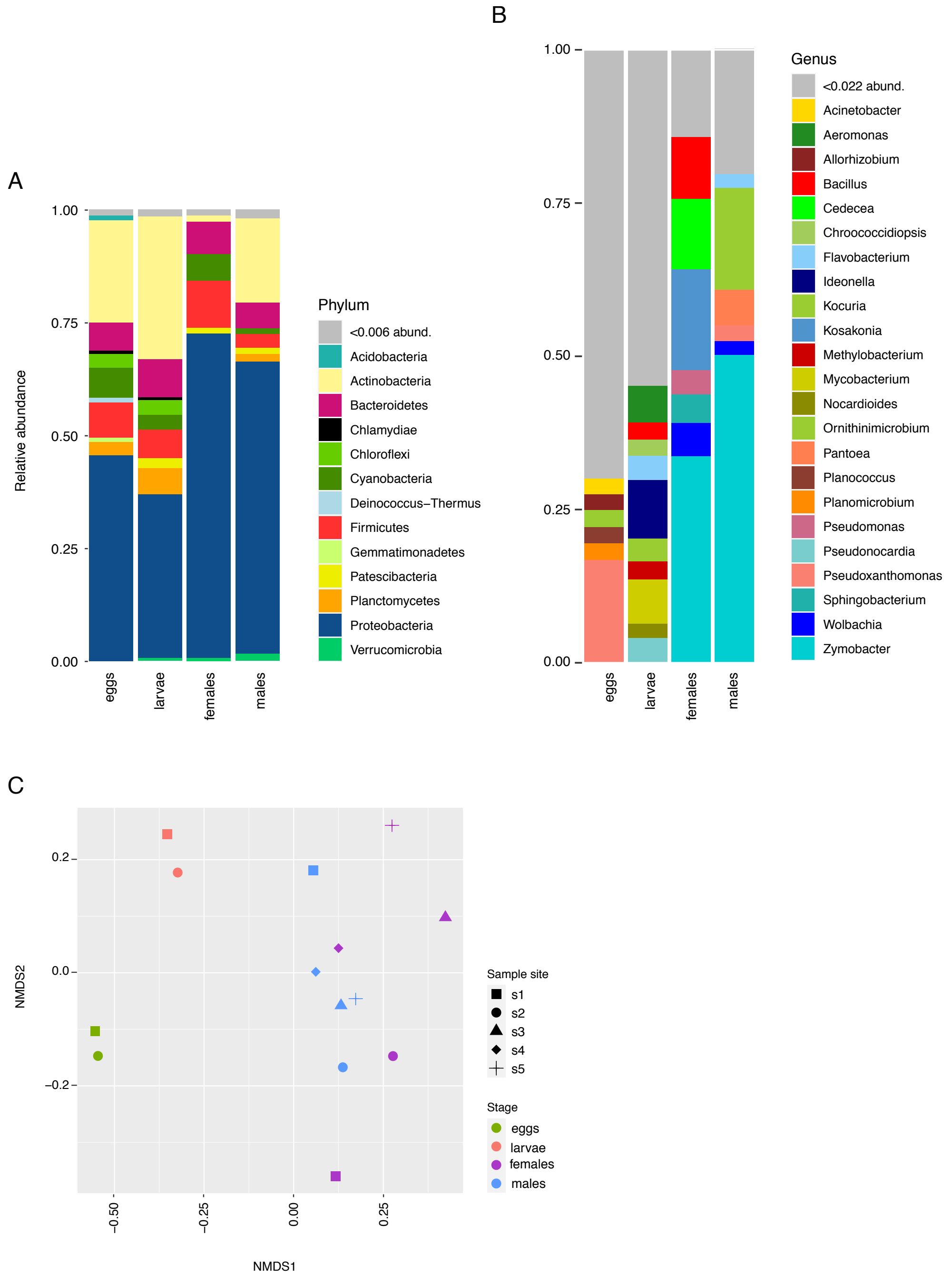

Fig. S2 ASVs diversity. A. Phyla distribution B. Genera distribution C. Non-metric multidimensional scaling (NMDS).

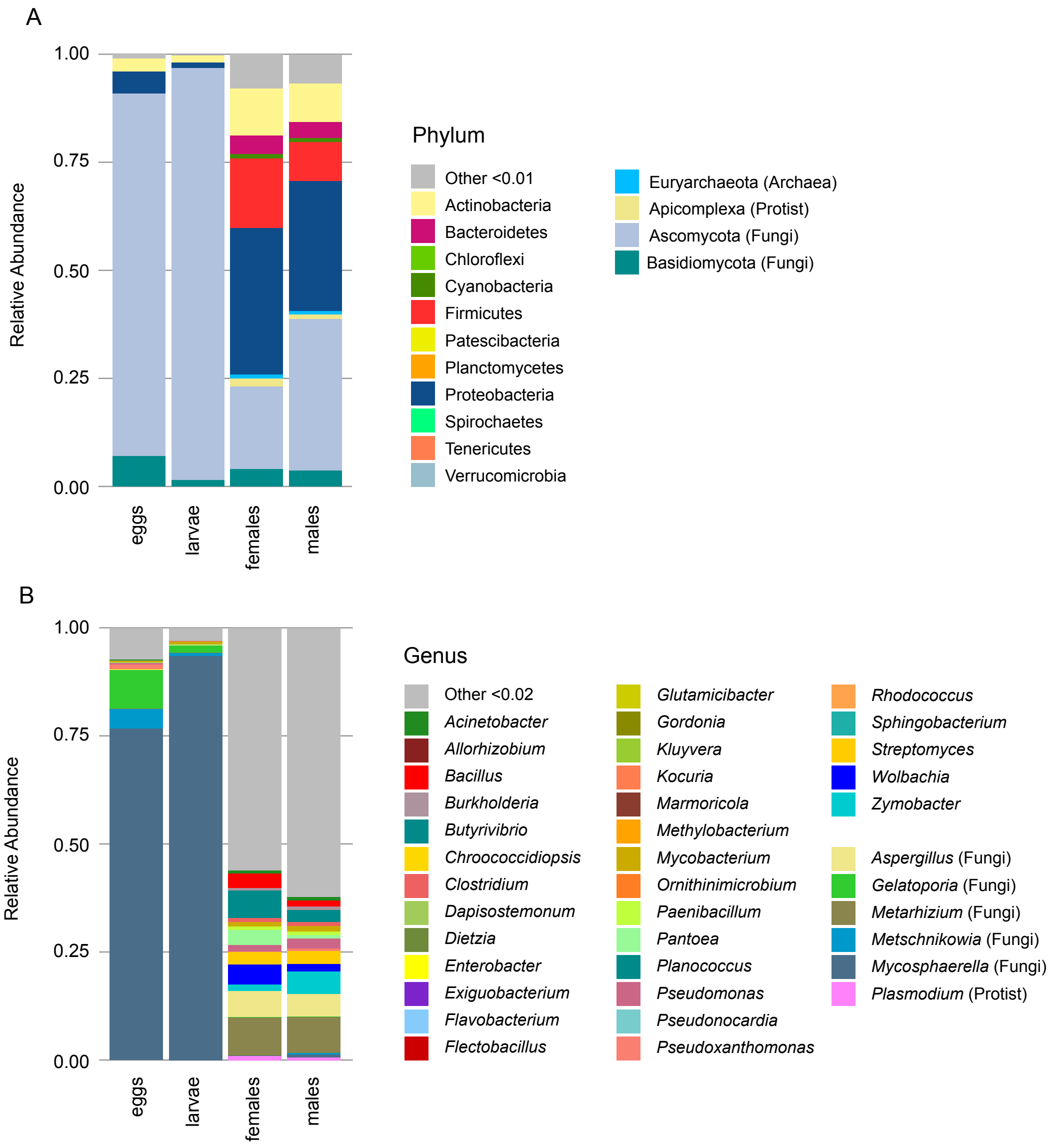

Fig. S3 Phylogenetic profile of *Ae. aegypti* microbiome from shotgun sequences. A. Observed phyla. B. Observed genera.

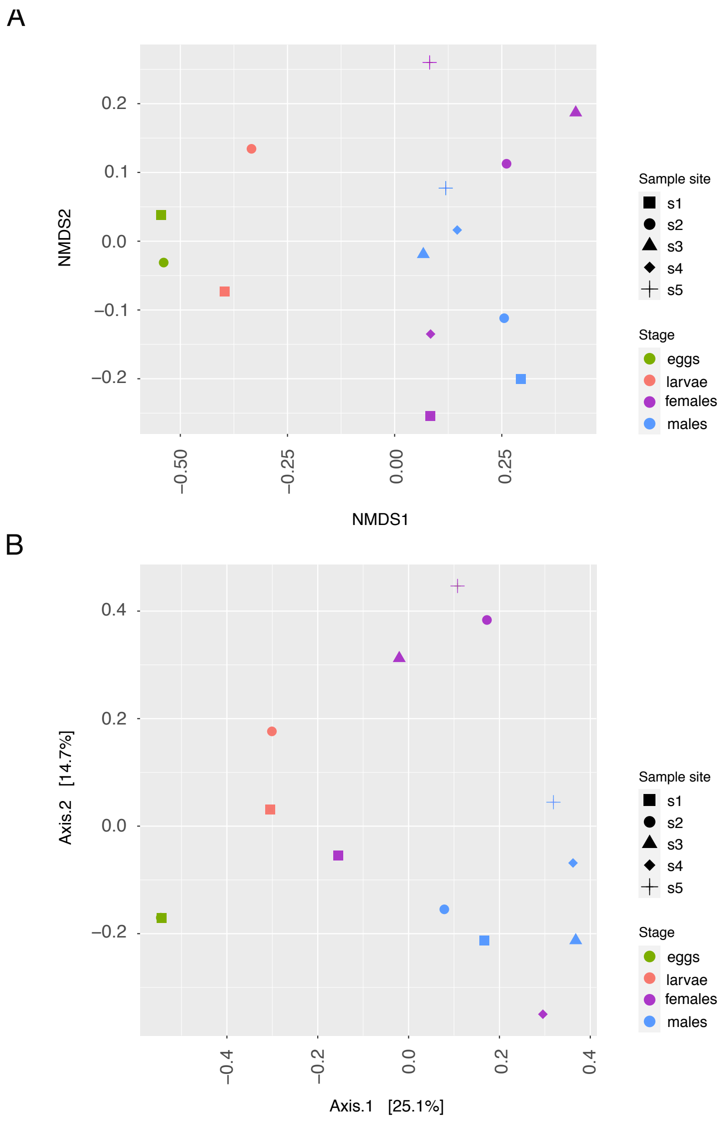

Fig. S4 Beta diversity plots for OTUs. A. Non-metric multidimensional scaling (NMDS) B. Principal Component Analysis (PCA).

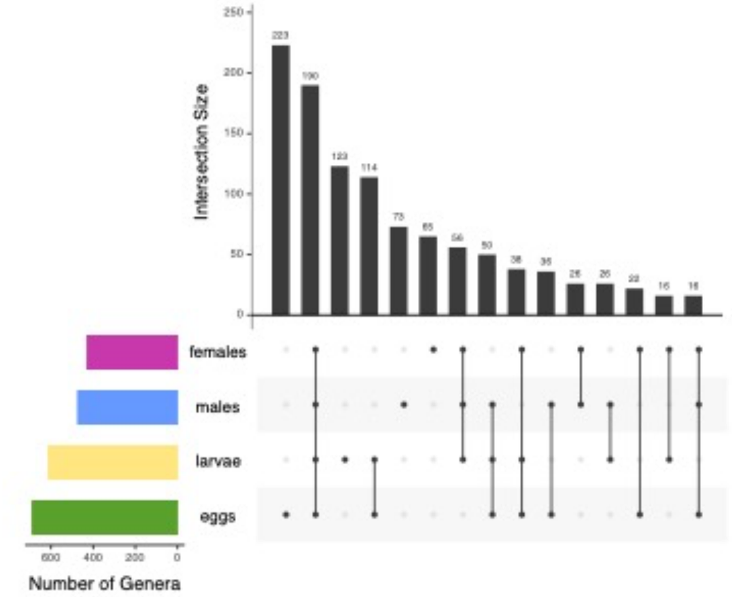

Fig. S5 OTUs Upset plot.

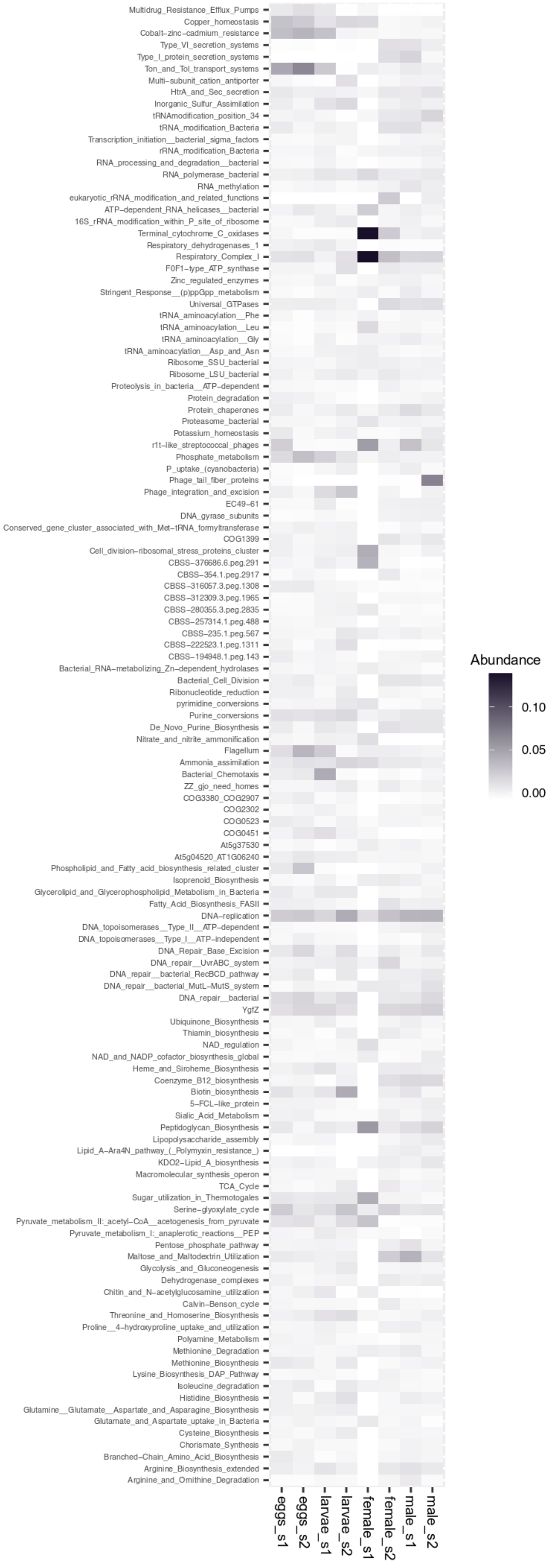

Fig. S6 Metabolic pathways abundances from shotgun sequences. Only pathways with an abundance greater than 0.2% are shown.

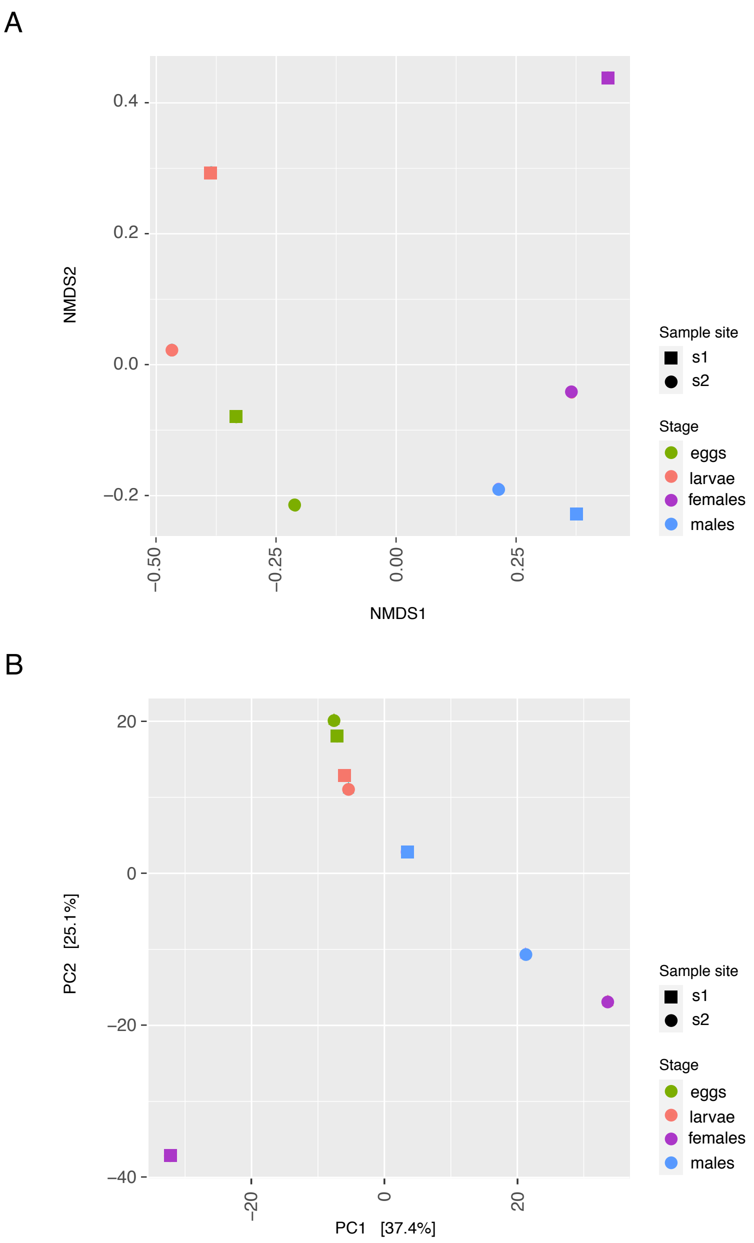

Fig. S7 Beta diversity plots for shotgun sequences. A. Non-metric multidimensional scaling (NMDS) B. Principal Component Analysis (PCA).

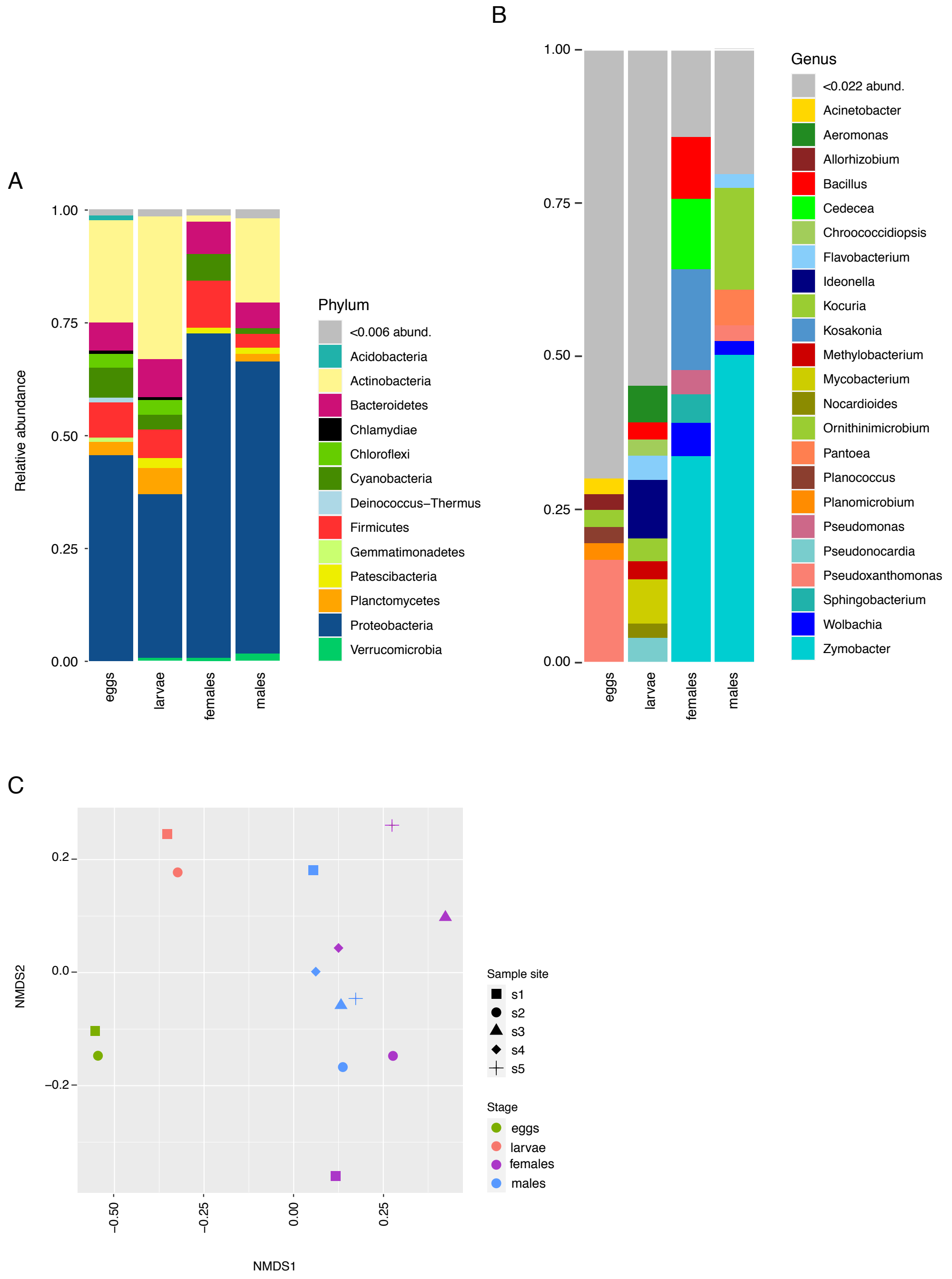

Fig. S8 ASVs diversity. A. Phyla distribution B. Genera distribution C. Non-metric multidimensional scaling (NMDS).

Table S1. Samples metadata.

Sample Name	Site key	Town Name	Collection Date	GPS coordinates	Altitude (m)	temperature (C)	material	stage	size (individuals)	16S amplicon sequencing	shotgun sequencing
eggs_s1	s1	Tlaquitenango	2018-06-06	18.6236 N 99.1578 W	863	29.6	all	eggs	100-200	yes	yes
eggs_s2	s2	Jojutla	2018-06-06	18.6089 N 99.1836 W	864	32.3	all	eggs	100-200	yes	yes
larves_s1	s1	Tlaquitenango	2018-06-26	18.6236 N 99.1578 W	863	29.6	gut	larvae	30	yes	yes
larves_s2	s2	Jojutla	2018-07-10	18.6089 N 99.1836 W	864	32.3	gut	larvae	30	yes	yes
females_s1	s1	Tlaquitenango	2018-06-26	18.6236 N 99.1578 W	863	29.6	gut	females	30	yes	yes
females_s2	s2	Jojutla	2018-07-10	18.6089 N 99.1836 W	864	32.3	gut	females	30	yes	yes
females_s3	s3	Tlaltizapan	2018-06-26	18.6865 N 99.1177 W	869	25.3	gut	females	30	yes	no
females_s4	s4	Puente_Ixtla	2018-07-03	18.6247 N 99.3239 W	917	31.4	gut	females	30	yes	no
females_s5	s5	Amacuzac	2018-07-10	18.6030 N 99.3819 W	865	26.5	gut	females	30	yes	no
males_s1	s1	Tlaquitenango	2018-06-26	18.6236 N 99.1578 W	863	29.6	gut	males	30	yes	yes
males_s2	s2	Jojutla	2018-07-10	18.6089 N 99.1836 W	864	32.3	gut	males	30	yes	yes
males_s3	s3	Tlaltizapan	2018-06-26	18.6865 N 99.1177 W	869	25.3	gut	males	30	yes	no
males_s4	s4	Puente_Ixtla	2018-07-03	18.6247 N 99.3239 W	917	31.4	gut	males	30	yes	no
males_s5	s5	Amacuzac	2018-07-10	18.6030 N 99.3819 W	865	26.5	gut	males	30	yes	no

Table S2. Sequencing effort and reads assignment summary.

	16S amplicons				Shotgun reads							
	Amplicon	After filtering	OTUs	ASVs	Shotgun	After filtering	% Aedes	Contigs	N50	Proteins	assigned	% bacteria
eggs_s1	305705	116883	2818	907	21500254	18833775	9.49	89450	231	91265	56417	97.68
eggs_s2	325224	143063	2734	1127	23936490	18029772	21.88	247357	261	264114	156273	98.06
larves_s1	409018	104791	2256	901	4137138	931116	75.79	44090	240	40985	19123	95.06
larves_s2	270404	127367	1107	461	19834418	17963054	6.6	51348	245	50481	31278	97.98
females_s1	316040	116101	675	194	34536410	7212655	77.39	90450	267	70600	2470	38.33
females_s2	223609	111370	588	71	46430024	23340717	47.33	706600	268	546119	29558	27.27
females_s3	267705	151681	523	117	na	na	na	na	na	na	na	na
females_s4	255134	124579	722	270	na	na	na	na	na	na	na	na
females_s5	291840	156889	605	118	na	na	na	na	na	na	na	na
males_s1	352993	133437	701	459	53821496	4543492	90.87	168325	256	123102	11357	60.41
males_s2	232500	127392	804	185	47843630	22263067	51.17	756988	243	605087	24846	20.82
males_s3	284448	164106	1067	278	na	na	na	na	na	na	na	na
males_s4	343550	196905	1059	371	na	na	na	na	na	na	na	na
males_s5	214364	107957	771	169	na	na	na	na	na	na	na	na

Table S3. Alpha diversity indexes for OTUs.

Sample	OTUs	Chao1	se.chao1	ACE	se.ACE	Shannon	Simpson	InvSimpson	Fisher
eggs_s1	2818	3129.55	33.20	3270.90	27.16	4.76	0.95	21.31	519.96
eggs_s2	2734	3039.37	34.19	3132.48	26.93	4.87	0.96	25.59	479.51
larves_s1	2256	2344.22	14.57	2414.46	22.78	5.05	0.98	44.23	405.95
larves_s2	1107	1173.56	13.64	1230.95	17.21	4.13	0.96	22.89	166.72
females_s1	675	722.15	11.21	778.73	14.22	3.08	0.81	5.23	94.94
females_s2	588	1003.67	64.73	1145.53	21.35	1.81	0.73	3.66	81.42
females_s3	523	638.78	22.98	707.17	14.18	2.14	0.82	5.59	67.80
females_s4	722	1013.33	46.03	1138.61	21.18	1.57	0.44	1.80	101.50
females_s5	605	866.55	43.94	946.89	17.37	1.52	0.70	3.28	79.77
males_s1	701	951.86	39.98	998.29	17.41	2.35	0.84	6.41	96.99
males_s2	804	922.96	21.27	990.76	15.03	1.37	0.47	1.88	114.62
males_s3	1067	1681.01	78.84	1785.68	24.75	2.06	0.69	3.26	152.87
males_s4	1059	1651.50	73.44	1969.94	29.34	2.53	0.81	5.24	147.08
males_s5	771	1167.26	59.90	1172.77	18.78	1.92	0.69	3.22	112.23

Table S4. Alpha diversity indexes for ASVs.

Samples	ASV	Sequences	Chao1	se.chao1	ACE	se.ACE	Shannon	Simpson	InvSimpson	Fisher
eggs_s1	907	35108	907.91	1.09	911.67	14.30	5.88	0.99	77.15	170.00
eggs_s2	1127	45634	1127.00	0.01	1127.31	14.79	6.18	0.99	101.76	209.08
larvae_s1	901	40338	901.05	0.23	901.82	13.84	5.59	0.98	47.74	163.44
larvae_s2	461	29331	461.00	0.04	461.36	6.17	5.22	0.98	66.49	77.65
females_s1	194	20217	194.20	0.53	195.09	6.32	3.27	0.90	9.58	29.74
females_s2	71	17388	71.00	0.00	71.00	3.52	2.40	0.81	5.38	9.44
females_s3	117	28626	117.00	0.00	117.00	4.72	2.90	0.91	11.24	15.56
females_s4	270	38629	270.00	0.00	270.00	3.88	3.90	0.94	16.85	39.16
females_s5	118	28163	118.00	0.00	118.00	5.11	2.94	0.92	12.59	15.76
males_s1	459	67463	459.25	0.74	459.98	3.58	4.36	0.97	30.48	66.27
males_s2	185	19857	185.00	0.00	185.00	6.22	3.31	0.91	10.68	28.21
males_s3	278	48249	278.00	0.10	278.16	6.01	3.53	0.93	15.30	39.04
males_s4	371	57564	371.60	1.19	372.43	4.15	4.19	0.96	24.02	53.08
males_s5	169	22819	169.14	0.49	169.37	6.09	3.13	0.90	9.70	24.75

Table S5. Phyla abundance from OTUs.

Phyla	Eggs (%)	Larvae (%)	Females (%)	Males (%)	Adults (%)	Aquatic (%)
Euryarchaeota	0.001	0.013	0.000	0.000	0.000	0.007
Ambiguous_taxa	0.001	0.003	0.000	0.001	0.000	0.002
Acidobacteria	0.536	0.158	0.100	0.079	0.089	0.347
Actinobacteria	18.482	41.891	10.187	1.070	5.629	30.187
Armatimonadetes	0.030	0.017	0.061	0.051	0.056	0.023
BRC1	0.012	0.000	0.000	0.000	0.000	0.006
Bacteroidetes	3.286	13.243	4.064	8.693	6.379	8.264
Chlamydiae	0.404	0.334	0.011	0.015	0.013	0.369
Chloroflexi	1.785	2.583	0.094	0.079	0.086	2.184
Cyanobacteria	6.562	4.559	0.059	0.004	0.031	5.560
Dadabacteria	0.000	0.001	0.000	0.000	0.000	0.000
Deinococcus-Thermus	0.454	0.154	0.013	0.002	0.008	0.304
Dependentiae	0.010	0.189	0.079	0.077	0.078	0.100
Epsilonbacteraeota	0.000	0.002	0.014	0.014	0.014	0.001
FBP	0.015	0.000	0.000	0.000	0.000	0.007
Fibrobacteres	0.001	0.000	0.000	0.000	0.000	0.000
Firmicutes	16.412	9.607	21.130	2.267	11.699	13.010
Fusobacteria	0.001	0.004	0.000	0.011	0.006	0.003
Gemmatimonadetes	0.443	0.056	0.006	0.016	0.011	0.250
Hydrogenedentes	0.015	0.003	0.000	0.000	0.000	0.009
Lentisphaerae	0.000	0.001	0.000	0.000	0.000	0.000
Nitrospirae	0.003	0.000	0.000	0.000	0.000	0.001
Patescibacteria	0.246	0.611	0.403	0.180	0.292	0.428
Planctomycetes	2.370	3.226	0.483	0.448	0.466	2.798
Proteobacteria	48.723	22.901	61.419	85.253	73.336	35.812
Spirochaetes	0.000	0.003	0.992	0.192	0.592	0.002
Tenericutes	0.000	0.001	0.002	1.163	0.582	0.000
Verrucomicrobia	0.121	0.410	0.880	0.384	0.632	0.266
WPS-2	0.088	0.030	0.001	0.000	0.001	0.059
WS4	0.000	0.000	0.001	0.000	0.000	0.000

Table S6. **Phyla abundance from metagenome shotgun sequences.**

Phylum	eggs_s1	%	eggs_s2	%	larves_s1	%	larves_s2	%	females_s	%	females_s	%	males_s1	%	males_s2	%
f_Ascomycota	5130421	78.3495	6322285	88.9863	2070	2.2535	5373976	96.8593	15275	14.6632	175713	19.6439	10746	9.3126	367915	38.1509
Proteobacteria	522550	7.98015	179661	2.52873	57020	62.0747	9983	0.17993	32015	30.7328	307294	34.354	65667	56.9078	258844	26.8408
f_Basidiomycota	443253	6.76916	504317	7.09827	567	0.61726	77895	1.40396	3834	3.68045	36012	4.02597	3114	2.69863	36604	3.79565
Actinobacteria	338859	5.1749	56000	0.7882	25759	28.0425	71232	1.28387	13627	13.0813	94247	10.5364	10100	8.75277	86875	9.0085
Cyanobacteria	16847	0.25728	2505	0.03526	368	0.40062	905	0.01631	1728	1.6588	9069	1.01387	1262	1.09366	8256	0.85611
Firmicutes	16146	0.24657	9233	0.12995	1867	2.03251	1457	0.02626	12258	11.7671	147701	16.5123	9074	7.86363	88912	9.21973
Bacteroidetes	13832	0.21124	2875	0.04047	1318	1.43484	626	0.01128	6420	6.16288	35768	3.99869	4829	4.18487	34050	3.53081
Acidobacteria	10750	0.16417	1034	0.01455	145	0.15785	81	0.00146	346	0.33214	2501	0.2796	323	0.27992	2195	0.22761
Mucoromycota	9671	0.14769	12547	0.1766	86	0.09362	8117	0.1463	899	0.863	5463	0.61074	483	0.41857	6231	0.64612
Planctomycetes	9207	0.14061	947	0.01333	473	0.51493	409	0.00737	753	0.72284	4564	0.51023	517	0.44804	4727	0.49017
Chloroflexi	6663	0.10175	844	0.01188	213	0.23188	823	0.01483	594	0.57021	3067	0.34288	490	0.42464	3286	0.34074
Euryarchaeota	3397	0.05188	2203	0.03101	220	0.2395	399	0.00719	1142	1.09626	7821	0.87435	926	0.80248	7151	0.74152
Nitrospirae	3208	0.04899	112	0.00158	65	0.07076	33	0.00059	217	0.20831	1261	0.14097	205	0.17766	1278	0.13252
Deinococcus-Thermus	3207	0.04898	599	0.00843	111	0.12084	169	0.00305	289	0.27743	3307	0.36971	176	0.15252	3388	0.35132
Gemmatimonadetes	2927	0.0447	427	0.00601	70	0.07621	19	0.00034	123	0.11807	1021	0.11414	88	0.07626	858	0.08897
Verrucomicrobia	2145	0.03276	230	0.00324	119	0.12955	56	0.00101	525	0.50397	2207	0.24673	310	0.26865	2788	0.2891
P_Apicomplexa	2097	0.03202	1740	0.02449	326	0.3549	302	0.00544	6071	5.82786	11792	1.31829	1849	1.60236	8947	0.92776
Tenericutes	1409	0.02152	1668	0.02348	28	0.03048	196	0.00353	397	0.3811	2119	0.23689	322	0.27905	2191	0.2272
Chlorophyta	896	0.01368	182	0.00256	109	0.11866	77	0.00139	539	0.51741	3940	0.44047	325	0.28165	3567	0.36988
Microsporidia	835	0.01275	1136	0.01599	21	0.02286	372	0.0067	261	0.25055	2592	0.28977	181	0.15686	2621	0.27178
Spirochaetes	781	0.01193	144	0.00203	107	0.11649	75	0.00135	839	0.8054	3892	0.43511	604	0.52343	3666	0.38015
Candidatus_Rokubacteria	757	0.01156	62	0.00087	22	0.02395	18	0.00032	68	0.06528	413	0.04617	44	0.03813	388	0.04023
Chytridiomycota	570	0.0087	604	0.0085	23	0.02504	340	0.00613	265	0.25439	1924	0.21509	180	0.15599	1999	0.20729
Armatimonadetes	468	0.00715	53	0.00075	19	0.02068	25	0.00045	50	0.048	969	0.10833	53	0.04593	1120	0.11614
Fusobacteria	446	0.00681	21	0.0003	27	0.02939	10	0.00018	236	0.22655	1955	0.21856	151	0.13086	1566	0.16239
Candidatus_Tectomicrobia	408	0.00623	108	0.00152	10	0.01089	36	0.00065	26	0.02496	329	0.03678	25	0.02167	314	0.03256
Elusimicrobia	319	0.00487	41	0.00058	34	0.03701	14	0.00025	123	0.11807	746	0.0834	111	0.09619	699	0.07248
Candidatus_Omnitrophica	305	0.00466	38	0.00053	37	0.04028	9	0.00016	218	0.20927	914	0.10218	114	0.09879	972	0.10079
Ignavibacteriae	271	0.00414	118	0.00166	8	0.00871	34	0.00061	75	0.072	1006	0.11247	68	0.05893	923	0.09571
Lentisphaerae	269	0.00411	34	0.00048	11	0.01198	12	0.00022	91	0.08736	484	0.05411	79	0.06846	462	0.04791
Neocallimastigomycota	242	0.0037	302	0.00425	27	0.02939	113	0.00204	149	0.14303	921	0.10296	146	0.12653	840	0.0871
Chlamydiae	234	0.00357	146	0.00205	19	0.02068	34	0.00061	656	0.62973	1691	0.18905	118	0.10226	861	0.08928
Thaumarchaeota	220	0.00336	1359	0.01913	8	0.00871	6	0.00011	65	0.0624	876	0.09793	86	0.07453	1623	0.1683
Bacillariophyta	217	0.00331	71	0.001	51	0.05552	19	0.00034	580	0.55677	2449	0.27379	247	0.21405	1837	0.19049
Chlorobi	209	0.00319	23	0.00032	13	0.01415	10	0.00018	85	0.0816	523	0.05847	53	0.04593	447	0.04635
Crenarchaeota	208	0.00318	49	0.00069	32	0.03484	22	0.0004	194	0.18623	1268	0.14176	177	0.15339	1163	0.1206
Candidatus_Woesebacteria	205	0.00313	211	0.00297	9	0.0098	28	0.0005	127	0.12191	354	0.03958	72	0.0624	333	0.03453
Candidatus_Saccharibacteria	186	0.00284	24	0.00034	11	0.01198	8	0.00014	20	0.0192	141	0.01576	21	0.0182	112	0.01161
Thermotogae	152	0.00232	22	0.00031	21	0.02286	15	0.00027	171	0.16415	765	0.08552	131	0.11353	721	0.07476
Zoopagomycota	148	0.00226	201	0.00283	24	0.02613	21	0.00038	341	0.32734	1898	0.21219	224	0.19412	1738	0.18022
Synergistetes	147	0.00224	20	0.00028	23	0.02504	12	0.00022	70	0.0672	401	0.04483	56	0.04853	363	0.03764
Blastocladiomycota	143	0.00218	20	0.00028	14	0.01524	11	0.0002	60	0.0576	1646	0.18402	57	0.0494	1102	0.11427
candidate_division_NC10	131	0.002	13	0.00018	7	0.00762	5	9E-05	15	0.0144	116	0.01297	17	0.01473	123	0.01275
Candidatus_Aminicenantes	126	0.00192	16	0.00023	8	0.00871	4	7.2E-05	32	0.03072	149	0.01666	33	0.0286	149	0.01545
Phaeophyceae	98	0.0015	11	0.00015	18	0.0196	6	0.00011	62	0.05952	370	0.04136	54	0.0468	448	0.04646
Balneolaeota	93	0.00142	20	0.00028	6	0.00653	8	0.00014	17	0.01632	108	0.01207	36	0.0312	137	0.01421
Candidatus_Handelsmanbacteria	92	0.0014	8	0.00011	4	0.00435	2	3.6E-05	2	0.00192	35	0.00391	3	0.0026	40	0.00415
Aquificae	89	0.00136	12	0.00017	8	0.00871	5	9E-05	46	0.04416	495	0.05534	58	0.05026	369	0.03826
candidate_division_Zixibacteria	86	0.00131	10	0.00014	11	0.01198	7	0.00013	27	0.02592	234	0.02616	27	0.0234	223	0.02312
Chromerida	73	0.00111	10	0.00014	14	0.01524	12	0.00022	138	0.13247	536	0.05992	48	0.0416	455	0.04718
Fibrobacteres	67	0.00102	13	0.00018	6	0.00653	3	5.4E-05	56	0.05376	486	0.05433	32	0.02773	427	0.04428
Candidatus_Kaiserbacteria	65	0.00099	14	0.0002	10	0.01089	9	0.00016	32	0.03072	165	0.01845	35	0.03033	173	0.01794
Candidatus_Peregrinibacteria	63	0.00096	4	5.6E-05	5	0.00544	4	7.2E-05	44	0.04224	179	0.02001	40	0.03466	240	0.02489
Nitrospinae	61	0.00093	10	0.00014	3	0.00327	3	5.4E-05	34	0.03264	178	0.0199	27	0.0234	116	0.01203
Candidatus_Gottesmanbacteria	58	0.00089	12	0.00017	14	0.01524	3	5.4E-05	88	0.08448	285	0.03186	42	0.0364	318	0.03297
Candidatus_Bathyarchaeota	55	0.00084	7	9.9E-05	2	0.00218	8	0.00014	41	0.03936	192	0.02146	28	0.02427	222	0.02302
Candidatus_Uhrbacteria	55	0.00084	3	4.2E-05	3	0.00327	1	1.8E-05	8	0.00768	197	0.02202	15	0.013	139	0.01441
Candidatus_Roizmanbacteria	52	0.00079	5	7E-05	5	0.00544	6	0.00011	65	0.0624	286	0.03197	43	0.03726	322	0.03339
Candidatus_Levybacteria	50	0.00076	20	0.00028	7	0.00762	4	7.2E-05	107	0.10271	347	0.03879	48	0.0416	295	0.03059
Candidatus_Eisenbacteria	49	0.00075	5	7E-05	2	0.00218	0	0	19	0.01824	45	0.00503	13	0.01127	40	0.00415
Candidatus_Dadabacteria	49	0.00075	1	1.4E-05	0	0	0	0	5	0.0048	55	0.00615	4	0.00347	44	0.00456
Candidatus_Cloacimonetes	49	0.00075	6	8.4E-05	4	0.00435	4	7.2E-05	6	0.00576	189	0.02113	17	0.01473	141	0.01462
Candidatus_Atribacteria	49	0.00075	66	0.00093	0	0	7	0.00013	15	0.0144	44	0.00492	4	0.00347	51	0.00529
Candidatus_Parcubacteria	48	0.00073	23	0.00032	20	0.02177	5	9E-05	234	0.22463	284	0.03175	86	0.07453	202	0.02095
Candidatus_Magasanikbacteria	47	0.00072	10	0.00014	6	0.00653	5	9E-05	37	0.03552	148	0.01655	33	0.0286	219	0.02271
Deferribacteres	46	0.0007	1	1.4E-05	5	0.00544	6	0.00011	33	0.03168	171	0.01912	25	0.02167	139	0.01441
Eustigmatophyceae	41	0.00063	4	5.6E-05	11	0.01198	4	7.2E-05	35	0.0336	228	0.02549	32	0.02773	220	0.02281
Candidatus_Latescibacteria	38	0.00058	4	5.6E-05	2	0.00218	1	1.8E-05	4	0.00384	59	0.0066	9	0.0078	44	0.00456
Candidatus_Schekmanbacteria	36	0.00055	4	5.6E-05	5	0.00544	0	0	4	0.00384	75	0.00838	15	0.013	94	0.00975
Thermodesulfobacteria	36	0.00055	5	7E-05	5	0.00544	1	1.8E-05	21	0.02016	252	0.02817	19	0.01647	192	0.01991
candidate_division_WWE3	34	0.00052	5	7E-05	2	0.00218	8	0.00014	40	0.0384	223	0.02493	24	0.0208	204	0.02115
Candidatus_Nomurabacteria	33	0.0005	11	0.00015	1	0.00109	2	3.6E-05	25	0.024	236	0.02638	18	0.0156	198	0.02053
Candidatus_Acetothermia	32	0.00049	7	9.9E-05	0	0	0	0	2	0.00192	163	0.01822	6	0.0052	144	0.01493
Candidatus_Marinimicrobia	32	0.00049	32	0.00045	2	0.00218	20	0.00036	23	0.02208	62	0.00693	15	0.013	44	0.00456
Candidatus_Amesbacteria	31	0.00047	3	4.2E-05	1	0.00109	0	0	18	0.01728	76	0.0085	13	0.01127	66	0.00684
Candidatus_Yanofskybacteria	28	0.00043	17	0.00024	10	0.01089	3	5.4E-05	55	0.0528	139	0.01554	22	0.01907	149	0.01545
Candidatus_Glassbacteria	27	0.00041	4	5.6E-05	0	0	1	1.8E-05	3	0.00288	42	0.0047	3	0.0026	35	0.00363
Candidatus_Colwellbacteria	27	0.00041	1	1.4E-05	23	0.02504	4	7.2E-05	171	0.16415	173	0.01934	109	0.09446	146	0.01514
Candidatus_Curtissbacteria	26	0.0004	10	0.00014	3	0.00327	0	0	49	0.04704	171	0.01912	26	0.02253	156	0.01618
Candidatus_Daviesbacteria	25	0.00038	7	9.9E-05	6	0.00653	7	0.00013	33	0.03168	239	0.02672	30	0.026	242	0.02509
Calditrichaeota	24	0.00037	1	1.4E-05	1	0.00109	0	0	0	0	79	0.00883	8	0.00693	53	0.0055
Candidatus_Moranbacteria	23	0.00035	12	0.00017	3	0.00327	2	3.6E-05	50	0.048	315	0.03522	17	0.01473	247	0.02561
Candidatus_Falkowbacteria	22	0.00034	10	0.00014	1	0.00109	3	5.4E-05	92	0.08832	160	0.01789	19	0.01647	87	0.00902
Candidatus_Woykebacteria	20	0.00031	1	1.4E-05	3	0.00327	1	1.8E-05	1	0.00096	54	0.00604	7	0.00607	60	0.00622
Candidatus_Doudnabacteria	20	0.00031	6	8.4E-05	0	0	2	3.6E-05	12	0.01152	98	0.01096	5	0.00433	60	0.00622
Candidatus_Lindowbacteria	20	0.00031	4	5.6E-05	1	0.00109	0	0	3	0.00288	63	0.00704	1	0.00087	54	0.0056

Candidatus_Margulisbacteria	20	0.00031	3	4.2E-05	1	0.00109	1	1.8E-05	3	0.00288	52	0.00581	3	0.0026	29	0.00301
Candidatus_Firestonebacteria	19	0.00029	4	5.6E-05	3	0.00327	0	0	18	0.01728	72	0.00805	15	0.013	47	0.00487
Candidatus_Desantisbacteria	19	0.00029	3	4.2E-05	1	0.00109	1	1.8E-05	12	0.01152	39	0.00436	5	0.00433	35	0.00363
Chrysiogenetes	18	0.00027	10	0.00014	8	0.00871	0	0	0	0	39	0.00436	6	0.0052	36	0.00373
Candidatus_Thorarchaeota	18	0.00027	1	1.4E-05	1	0.00109	4	7.2E-05	6	0.00576	75	0.00838	12	0.0104	64	0.00664
Candidatus_Giovannonibacteria	18	0.00027	1	1.4E-05	4	0.00435	0	0	8	0.00768	71	0.00794	20	0.01733	94	0.00975
candidate_division_WOR-3	17	0.00026	3	4.2E-05	7	0.00762	1	1.8E-05	4	0.00384	71	0.00794	9	0.0078	51	0.00529
Candidatus_Riflebacteria	17	0.00026	0	0	2	0.00218	0	0	3	0.00288	16	0.00179	4	0.00347	23	0.00238
Candidatus_Ryanbacteria	16	0.00024	2	2.8E-05	4	0.00435	2	3.6E-05	1	0.00096	23	0.00257	2	0.00173	36	0.00373
Candidatus_Berkelbacteria	15	0.00023	4	5.6E-05	10	0.01089	1	1.8E-05	19	0.01824	82	0.00917	21	0.0182	85	0.00881
Candidatus_Taylorbacteria	15	0.00023	1	1.4E-05	0	0	3	5.4E-05	4	0.00384	59	0.0066	18	0.0156	57	0.00591
Candidatus_Lloydbacteria	15	0.00023	0	0	0	0	0	0	1	0.00096	60	0.00671	14	0.01213	58	0.00601
Candidatus_Sungbacteria	14	0.00021	0	0	0	0	0	0	12	0.01152	66	0.00738	7	0.00607	77	0.00798
Candidatus_Raymondbacteria	14	0.00021	1	1.4E-05	0	0	0	0	0	0	56	0.00626	8	0.00693	44	0.00456
Candidatus_Micrarchaeota	14	0.00021	3	4.2E-05	3	0.00327	4	7.2E-05	12	0.01152	63	0.00704	7	0.00607	59	0.00612
Candidatus_Buchananbacteria	14	0.00021	2	2.8E-05	1	0.00109	1	1.8E-05	67	0.06432	65	0.00727	13	0.01127	77	0.00798
Kiritimatiellaeota	13	0.0002	2	2.8E-05	2	0.00218	1	1.8E-05	1	0.00096	6	0.00067	1	0.00087	11	0.00114
Candidatus_Kryptonia	13	0.0002	2	2.8E-05	3	0.00327	1	1.8E-05	1	0.00096	40	0.00447	5	0.00433	74	0.00767
Candidatus_Lokiarchaeota	12	0.00018	1	1.4E-05	2	0.00218	0	0	50	0.048	86	0.00961	9	0.0078	89	0.00923
Candidatus_Collierbacteria	12	0.00018	2	2.8E-05	0	0	0	0	54	0.05184	95	0.01062	12	0.0104	54	0.0056
Candidatus_Adlerbacteria	12	0.00018	0	0	1	0.00109	0	0	5	0.0048	24	0.00268	1	0.00087	25	0.00259
Candidatus_Wildermuthbacteria	11	0.00017	0	0	0	0	0	0	19	0.01824	67	0.00749	5	0.00433	65	0.00674
Candidatus_Wolfebacteria	11	0.00017	2	2.8E-05	4	0.00435	0	0	15	0.0144	219	0.02448	8	0.00693	97	0.01006
Candidatus_Spechtbacteria	11	0.00017	3	4.2E-05	0	0	1	1.8E-05	5	0.0048	23	0.00257	3	0.0026	18	0.00187
Candidatus_Nanohaloarchaeota	11	0.00017	6	8.4E-05	1	0.00109	0	0	4	0.00384	38	0.00425	5	0.00433	51	0.00529
Candidatus_Nealsonbacteria	11	0.00017	0	0	1	0.00109	3	5.4E-05	2	0.00192	31	0.00347	10	0.00867	35	0.00363
Candidatus_Fischerbacteria	11	0.00017	2	2.8E-05	0	0	0	0	0	0	15	0.00168	2	0.00173	47	0.00487
Candidatus_Coatesbacteria	11	0.00017	0	0	2	0.00218	1	1.8E-05	3	0.00288	8	0.00089	1	0.00087	13	0.00135
Dictyoglomi	10	0.00015	2	2.8E-05	1	0.00109	0	0	11	0.01056	51	0.0057	6	0.0052	40	0.00415
Candidatus_Staskawiczbacteria	10	0.00015	4	5.6E-05	1	0.00109	3	5.4E-05	14	0.01344	120	0.01342	2	0.00173	74	0.00767
Candidatus_Portnoybacteria	10	0.00015	1	1.4E-05	1	0.00109	0	0	3	0.00288	11	0.00123	5	0.00433	23	0.00238
Candidatus_Edwardsbacteria	10	0.00015	0	0	1	0.00109	1	1.8E-05	0	0	19	0.00212	4	0.00347	16	0.00166
Candidatus_Beckwithbacteria	10	0.00015	3	4.2E-05	1	0.00109	0	0	7	0.00672	54	0.00604	7	0.00607	52	0.00539
candidate_division_KD3-62	10	0.00015	1	1.4E-05	0	0	0	0	0	0	0	0	0	0	7	0.00073
candidate_division_Hyd24-12	10	0.00015	1	1.4E-05	0	0	0	0	17	0.01632	38	0.00425	2	0.00173	23	0.00238
Candidatus_Shapirobacteria	9	0.00014	2	2.8E-05	1	0.00109	3	5.4E-05	14	0.01344	50	0.00559	13	0.01127	38	0.00394
Candidatus_Terrybacteria	9	0.00014	0	0	0	0	0	0	13	0.01248	23	0.00257	6	0.0052	31	0.00321
Candidatus_Blackburnbacteria	9	0.00014	3	4.2E-05	1	0.00109	0	0	23	0.02208	39	0.00436	6	0.0052	21	0.00218
Cryptomycota	9	0.00014	0	0	4	0.00435	3	5.4E-05	19	0.01824	362	0.04047	14	0.01213	283	0.02935
Candidatus_Jorgensenbacteria	9	0.00014	1	1.4E-05	5	0.00544	1	1.8E-05	17	0.01632	43	0.00481	9	0.0078	41	0.00425
Candidatus_Zambryskibacteria	8	0.00012	3	4.2E-05	2	0.00218	0	0	16	0.01536	44	0.00492	16	0.01387	47	0.00487
Candidatus_Azambacteria	8	0.00012	7	9.9E-05	0	0	2	3.6E-05	10	0.0096	60	0.00671	7	0.00607	54	0.0056
Candidatus_Yonathbacteria	8	0.00012	1	1.4E-05	4	0.00435	0	0	3	0.00288	28	0.00313	0	0	31	0.00321
Candidatus_Pacebacteria	7	0.00011	2	2.8E-05	1	0.00109	6	0.00011	4	0.00384	50	0.00559	6	0.0052	44	0.00456
Candidatus_Wallbacteria	7	0.00011	5	7E-05	1	0.00109	0	0	3	0.00288	83	0.00928	2	0.00173	57	0.00591
Candidatus_Chisholmbacteria	7	0.00011	1	1.4E-05	1	0.00109	3	5.4E-05	3	0.00288	20	0.00224	3	0.0026	21	0.00218
Caldiserica	7	0.00011	2	2.8E-05	0	0	1	1.8E-05	5	0.0048	25	0.00279	6	0.0052	28	0.0029
Candidatus_Hydrogenedentes	7	0.00011	0	0	1	0.00109	1	1.8E-05	1	0.00096	29	0.00324	3	0.0026	17	0.00176
candidate_division_CPR3	7	0.00011	1	1.4E-05	3	0.00327	1	1.8E-05	1	0.00096	37	0.00414	2	0.00173	35	0.00363
Candidatus_Andersenbacteria	6	9.2E-05	0	0	0	0	0	0	1	0.00096	22	0.00246	3	0.0026	19	0.00197
Candidatus_Liptonbacteria	6	9.2E-05	0	0	0	0	0	0	3	0.00288	42	0.0047	0	0	28	0.0029
Candidatus_Kerfeldbacteria	6	9.2E-05	0	0	1	0.00109	0	0	6	0.00576	29	0.00324	6	0.0052	27	0.0028
Candidatus_Harrisonbacteria	6	9.2E-05	0	0	1	0.00109	1	1.8E-05	2	0.00192	27	0.00302	4	0.00347	17	0.00176
Candidatus_Komeilibacteria	6	9.2E-05	0	0	2	0.00218	0	0	3	0.00288	44	0.00492	8	0.00693	24	0.00249
Candidatus_Abawacabacteria	6	9.2E-05	0	0	0	0	0	0	0	0	25	0.00279	0	0	11	0.00114
Candidatus_Woesearchaeota	6	9.2E-05	1	1.4E-05	0	0	0	0	3	0.00288	30	0.00335	6	0.0052	40	0.00415
candidate_division_CPR1	5	7.6E-05	0	0	0	0	0	0	0	0	11	0.00123	0	0	10	0.00104
Candidatus_Microgenomates	5	7.6E-05	0	0	0	0	0	0	1	0.00096	14	0.00157	9	0.0078	37	0.00384
candidate_division_CPR2	5	7.6E-05	1	1.4E-05	1	0.00109	0	0	0	0	12	0.00134	2	0.00173	11	0.00114
Candidatus_Fraserbacteria	5	7.6E-05	8	0.00011	0	0	0	0	0	0	25	0.00279	6	0.0052	14	0.00145
Candidatus_Wirthbacteria	5	7.6E-05	2	2.8E-05	0	0	0	0	1	0.00096	4	0.00045	3	0.0026	18	0.00187
Candidatus_Campbellbacteria	4	6.1E-05	0	0	0	0	0	0	0	0	12	0.00134	2	0.00173	17	0.00176
Candidatus_Kuenenbacteria	4	6.1E-05	2	2.8E-05	1	0.00109	0	0	1	0.00096	21	0.00235	1	0.00087	18	0.00187
Euglenida	4	6.1E-05	8	0.00011	0	0	0	0	3	0.00288	15	0.00168	1	0.00087	17	0.00176
Candidatus_Parvarchaeota	3	4.6E-05	0	0	0	0	0	0	1	0.00096	71	0.00794	1	0.00087	58	0.00601
Candidatus_Odinarchaeota	2	3.1E-05	0	0	0	0	0	0	1	0.00096	7	0.00078	1	0.00087	9	0.00093
Candidatus_Korarchaeota	1	1.5E-05	1	1.4E-05	0	0	0	0	1	0.00096	22	0.00246	2	0.00173	12	0.00124
Candidatus_Delongbacteria	1	1.5E-05	0	0	0	0	1	1.8E-05	0	0	30	0.00335	2	0.00173	18	0.00187
Candidatus_Niyogibacteria	1	1.5E-05	0	0	1	0.00109	1	1.8E-05	0	0	8	0.00089	3	0.0026	8	0.00083
Nanoarchaeota	1	1.5E-05	0	0	1	0.00109	0	0	0	0	2	0.00022	4	0.00347	14	0.00145
Candidatus_Vogelbacteria	1	1.5E-05	0	0	0	0	0	0	0	0	6	0.00067	0	0	10	0.00104
Candidatus_Tagabacteria	1	1.5E-05	0	0	1	0.00109	0	0	0	0	6	0.00067	4	0.00347	14	0.00145
Candidatus_Gracilibacteria	1	1.5E-05	0	0	4	0.00435	0	0	5	0.0048	5	0.00056	9	0.0078	7	0.00073
Candidatus_Brennerbacteria	0	0	1	1.4E-05	0	0	0	0	0	0	10	0.00112	0	0	3	0.00031
Candidatus_Aenigmarchaeota	0	0	0	0	1	0.00109	0	0	0	0	9	0.00101	0	0	7	0.00073
Colponemidia	0	0	0	0	0	0	0	0	1	0.00096	7	0.00078	0	0	0	0
Xanthophyceae	0	0	0	0	0	0	0	0	0	0	2	0.00022	0	0	2	0.00021
Haplosporidia	0	0	0	0	0	0	0	0	0	0	1	0.00011	0	0	0	0
Candidatus_Poribacteria	0	0	0	0	0	0	0	0	0	0	1	0.00011	0	0	0	0
Candidatus_Jacksonbacteria	0	0	0	0	0	0	0	0	0	0	0	0	0	0	3	0.00031
All	6548125	100	7104786	100	91857	100	5548228	100	104172	100	894492	100	115392	100	964367	100

Table S7. Genera abundance from 16S sequences

genus					eggs_s1	eggs_s2	larves_s1	larves_s2	females_s1	females_s2	females_s3	females_s4	females_s5	males_s1	males_s2	males_s3	males_s4	males_s5	all
P_Proteobacteria	C_Gammaproteobacteri	O_Oceanospirillales	F_Halomonadaceae	G_Zymobacter	0.0003	0.0001	0.0004	0.0001	0.0179	0.2044	0.0121	0.8515	0.2226	0.2623	0.8252	0.7412	0.6610	0.7479	0.32480
P_Firmicutes	C_Bacilli	O_Bacillales	F_Bacillaceae	G_Bacillus	0.0115	0.0102	0.0021	0.0873	0.0001	0.4647	0.0000	0.0004	0.4615	0.0001	0.0000	0.0001	0.0003	0.0493	0.07769
P_Proteobacteria	C_Alphaproteobacteria	O_Rickettsiales	F_Anaplasmataceae	G_Wolbachia	0.0000	0.0000	0.0003	0.0000	0.0000	0.3049	0.3296	0.0004	0.0007	0.0000	0.0000	0.0000	0.1581	0.0000	0.05673
P_Proteobacteria	C_Gammaproteobacteri	O_Xanthomonadales	F_Xanthomonadaceae	G_Pseudoxanthomonas	0.2134	0.1900	0.0009	0.0004	0.0061	0.0000	0.0018	0.0014	0.0017	0.0001	0.0001	0.1546	0.0154	0.1254	0.05081
P_Actinobacteria	C_Actinobacteria	O_Micrococcales	F_Intrasporangiaceae	G_Ornithinimicrobium	0.0009	0.0012	0.0003	0.0001	0.4652	0.0001	0.0002	0.0000	0.0000	0.0000	0.0001	0.0001	0.0000	0.0002	0.03345
P_Proteobacteria	C_Gammaproteobacteri	O_Enterobacteriales	F_Enterobacteriaceae	Ambiguous_taxa	0.0000	0.0001	0.0000	0.0000	0.0034	0.0007	0.2101	0.0005	0.1989	0.0045	0.0054	0.0023	0.0004	0.0016	0.03057
P_Proteobacteria	C_Gammaproteobacteri	O_Enterobacteriales	F_Enterobacteriaceae	G_Enterobacter	0.0008	0.0006	0.0001	0.0000	0.0011	0.0001	0.3391	0.0013	0.0000	0.0099	0.0625	0.0062	0.0016	0.0022	0.03040
P_Proteobacteria	C_Gammaproteobacteri	O_Pseudomonadales	F_Pseudomonadaceae	G_Pseudomonas	0.0289	0.0291	0.0031	0.0026	0.0050	0.0002	0.0001	0.0051	0.0001	0.2994	0.0056	0.0014	0.0016	0.0020	0.02745
P_Bacteroidetes	C_Bacteroidia	O_Sphingobacteriales	F_Sphingobacteriaceae	G_Sphingobacterium	0.0034	0.0036	0.0000	0.0000	0.0002	0.0000	0.0000	0.0000	0.0000	0.3502	0.0000	0.0000	0.0000	0.0000	0.02553
P_Bacteroidetes	C_Bacteroidia	O_Flavobacteriales	F_Flavobacteriaceae	G_Flavobacterium	0.0051	0.0066	0.1002	0.0027	0.0400	0.0002	0.0025	0.0233	0.0017	0.0004	0.0072	0.0098	0.0239	0.0040	0.01626
P_Actinobacteria	C_Actinobacteria	O_Micrococcales	F_Micrococcaceae	G_Kocuria	0.0620	0.0609	0.0013	0.0849	0.0038	0.0000	0.0000	0.0037	0.0001	0.0002	0.0003	0.0026	0.0000	0.0011	0.01579
P_Actinobacteria	C_Actinobacteria	O_Corynebacteriales	F_Mycobacteriaceae	G_Mycobacterium	0.0060	0.0068	0.1315	0.0727	0.0003	0.0000	0.0000	0.0000	0.0003	0.0000	0.0000	0.0001	0.0000	0.0000	0.01554
P_Firmicutes	C_Bacilli	O_Bacillales	F_Planococcaceae	G_Planococcus	0.1059	0.0980	0.0000	0.0000	0.0000	0.0000	0.0000	0.0000	0.0000	0.0000	0.0000	0.0000	0.0000	0.0000	0.01458
P_Actinobacteria	C_Actinobacteria	O_Pseudonocardiales	F_Pseudonocardiaceae	G_Pseudonocardia	0.0044	0.0042	0.0095	0.1257	0.0000	0.0000	0.0001	0.0000	0.0000	0.0000	0.0000	0.0001	0.0000	0.0000	0.01029
P_Proteobacteria	C_Gammaproteobacteri	O_Pseudomonadales	F_Moraxellaceae	G_Acinetobacter	0.0280	0.0308	0.0029	0.0181	0.0216	0.0017	0.0007	0.0058	0.0032	0.0023	0.0185	0.0006	0.0030	0.0007	0.00984
P_Proteobacteria	C_Alphaproteobacteria	O_Rhizobiales	F_Rhizobiaceae	G_Allorhizobium-Neorhiz	0.0406	0.0423	0.0341	0.0011	0.0073	0.0000	0.0001	0.0035	0.0001	0.0004	0.0001	0.0003	0.0009	0.0000	0.00934
P_Cyanobacteria	C_Oxyphotobacteria	O_Nostocales	F_Chroococcidiopsacea	G_Chroococcidiopsis_P	0.0073	0.0088	0.0000	0.0899	0.0000	0.0000	0.0000	0.0000	0.0000	0.0000	0.0000	0.0001	0.0000	0.0000	0.00758
P_Firmicutes	C_Bacilli	O_Bacillales	F_Staphylococcaceae	G_Staphylococcus	0.0013	0.0017	0.0015	0.0022	0.0801	0.0002	0.0000	0.0051	0.0011	0.0000	0.0004	0.0001	0.0010	0.0000	0.00677
P_Proteobacteria	C_Gammaproteobacteri	O_Enterobacteriales	F_Enterobacteriaceae	G_Kluyvera	0.0000	0.0000	0.0001	0.0000	0.0016	0.0000	0.0000	0.0000	0.0882	0.0003	0.0001	0.0000	0.0000	0.0003	0.00647
P_Proteobacteria	C_Alphaproteobacteria	O_Rhodobacterales	F_Rhodobacteraceae	Ambiguous_taxa	0.0108	0.0105	0.0588	0.0079	0.0000	0.0000	0.0000	0.0000	0.0001	0.0000	0.0000	0.0000	0.0003	0.0000	0.00632
P_Actinobacteria	C_Actinobacteria	O_Corynebacteriales	F_Nocardiaceae	G_Rhodococcus	0.0034	0.0036	0.0274	0.0444	0.0001	0.0000	0.0000	0.0005	0.0000	0.0000	0.0001	0.0002	0.0003	0.0000	0.00572
P_Bacteroidetes	C_Bacteroidia	O_Cytophagales	F_Spirosomaceae	G_Flectobacillus	0.0000	0.0000	0.0789	0.0000	0.0000	0.0000	0.0000	0.0000	0.0000	0.0000	0.0000	0.0000	0.0000	0.0000	0.00564
P_Proteobacteria	C_Alphaproteobacteria	O_Rhizobiales	F_Beijerinckiaceae	G_Methylobacterium	0.0006	0.0006	0.0037	0.0689	0.0028	0.0001	0.0000	0.0000	0.0001	0.0000	0.0001	0.0001	0.0011	0.0000	0.00559
P_Bacteroidetes	C_Bacteroidia	O_Cytophagales	F_Cytophagaceae	G_Rhodocytophaga	0.0014	0.0014	0.0000	0.0000	0.0723	0.0000	0.0000	0.0000	0.0000	0.0000	0.0000	0.0000	0.0000	0.0000	0.00536
P_Actinobacteria	C_Actinobacteria	O_Corynebacteriales	F_Nocardiaceae	G_Gordonia	0.0015	0.0013	0.0044	0.0603	0.0000	0.0000	0.0001	0.0000	0.0000	0.0000	0.0000	0.0002	0.0000	0.0000	0.00485
P_Proteobacteria	C_Gammaproteobacteri	O_Betaproteobacteriales	F_Burkholderiaceae	Ambiguous_taxa	0.0089	0.0102	0.0095	0.0004	0.0207	0.0010	0.0002	0.0027	0.0002	0.0000	0.0010	0.0016	0.0060	0.0006	0.00450
P_Actinobacteria	C_Actinobacteria	O_Corynebacteriales	F_Dietziaceae	G_Dietzia	0.0243	0.0248	0.0054	0.0051	0.0000	0.0000	0.0000	0.0000	0.0001	0.0000	0.0000	0.0000	0.0000	0.0001	0.00427
P_Spirochaetes	C_Spirochaetia	O_Spirochaetales	F_Spirochaetaceae	G_uncultured	0.0000	0.0000	0.0000	0.0000	0.0000	0.0027	0.0462	0.0000	0.0000	0.0001	0.0000	0.0041	0.0000	0.0052	0.00416
P_Tenericutes	C_Mollicutes	O_Entomoplasmatales	F_Entomoplasmataceae	G_Mesoplasma	0.0000	0.0000	0.0000	0.0000	0.0001	0.0000	0.0000	0.0000	0.0000	0.0172	0.0404	0.0003	0.0000	0.0001	0.00415
P_Cyanobacteria	C_Oxyphotobacteria	O_Nostocales	F_Chroococcidiopsacea	G_Dapisostemonum_CC	0.0261	0.0258	0.0000	0.0000	0.0000	0.0000	0.0000	0.0000	0.0000	0.0000	0.0000	0.0000	0.0000	0.0000	0.00371
P_Proteobacteria	C_Gammaproteobacteri	O_Betaproteobacteriales	F_Burkholderiaceae	G_Hydrogenophaga	0.0028	0.0033	0.0429	0.0007	0.0000	0.0000	0.0000	0.0007	0.0000	0.0000	0.0001	0.0000	0.0008	0.0000	0.00365
P_Bacteroidetes	C_Bacteroidia	O_Flavobacteriales	F_Weeksellaceae	G_Chryseobacterium	0.0003	0.0004	0.0318	0.0000	0.0106	0.0056	0.0001	0.0007	0.0001	0.0001	0.0000	0.0010	0.0000	0.0002	0.00363
P_Actinobacteria	C_Actinobacteria	O_Micrococcales	F_Micrococcaceae	G_Glutamicibacter	0.0251	0.0230	0.0001	0.0001	0.0000	0.0001	0.0000	0.0002	0.0000	0.0000	0.0004	0.0002	0.0011	0.0000	0.00359
P_Firmicutes	C_Bacilli	O_Bacillales	F_Family_XII	G_Exiguobacterium	0.0235	0.0247	0.0001	0.0016	0.0000	0.0000	0.0000	0.0000	0.0000	0.0000	0.0000	0.0000	0.0002	0.0000	0.00358
P_Actinobacteria	C_Actinobacteria	O_Propionibacteriales	F_Nocardioidaceae	G_Marmoricola	0.0011	0.0012	0.0060	0.0404	0.0000	0.0000	0.0000	0.0003	0.0000	0.0000	0.0000	0.0000	0.0000	0.0000	0.00350
P_Actinobacteria	C_Actinobacteria	O_Propionibacteriales	F_Nocardioidaceae	G_Nocardioides	0.0065	0.0076	0.0221	0.0045	0.0032	0.0001	0.0000	0.0017	0.0001	0.0000	0.0001	0.0007	0.0003	0.0001	0.00337
P_Firmicutes	C_Bacilli	O_Bacillales	F_Planococcaceae	G_Lysinibacillus	0.0003	0.0003	0.0437	0.0000	0.0000	0.0000	0.0000	0.0000	0.0000	0.0000	0.0000	0.0000	0.0000	0.0000	0.00316
P_Proteobacteria	C_Alphaproteobacteria	O_Caulobacterales	F_Caulobacteraceae	G_Brevundimonas	0.0179	0.0201	0.0010	0.0030	0.0001	0.0000	0.0000	0.0000	0.0000	0.0000	0.0000	0.0000	0.0000	0.0000	0.00301
P_Firmicutes	C_Bacilli	O_Bacillales	F_Planococcaceae	G_Planomicrobium	0.0216	0.0198	0.0001	0.0000	0.0000	0.0001	0.0000	0.0000	0.0001	0.0000	0.0000	0.0000	0.0000	0.0000	0.00298
P_Proteobacteria	C_Gammaproteobacteri	O_Enterobacteriales	F_Enterobacteriaceae	G_Klebsiella	0.0000	0.0000	0.0000	0.0000	0.0001	0.0000	0.0399	0.0000	0.0000	0.0003	0.0001	0.0000	0.0000	0.0000	0.00289
P_Proteobacteria	C_Gammaproteobacteri	O_Vibrionales	F_Vibrionaceae	G_Vibrio	0.0000	0.0000	0.0000	0.0000	0.0000	0.0000	0.0000	0.0000	0.0000	0.0392	0.0000	0.0000	0.0000	0.0000	0.00280
P_Actinobacteria	C_Actinobacteria	O_Pseudonocardiales	F_Pseudonocardiaceae	G_Actinomycetospora	0.0009	0.0011	0.0342	0.0028	0.0000	0.0000	0.0000	0.0000	0.0000	0.0000	0.0000	0.0000	0.0000	0.0001	0.00279
P_Proteobacteria	C_Gammaproteobacteri	O_Xanthomonadales	F_Xanthomonadaceae	G_Stenotrophomonas	0.0131	0.0125	0.0022	0.0000	0.0036	0.0000	0.0000	0.0000	0.0000	0.0001	0.0000	0.0022	0.0000	0.0038	0.00267
P_Proteobacteria	C_Alphaproteobacteria	O_Sphingomonadales	F_Sphingomonadaceae	G_Sphingomonas	0.0040	0.0047	0.0007	0.0011	0.0157	0.0001	0.0000	0.0015	0.0002	0.0000	0.0001	0.0001	0.0031	0.0003	0.00227
P_Actinobacteria	C_Actinobacteria	O_Micrococcales	F_Micrococcaceae	G_Micrococcus	0.0015	0.0010	0.0000	0.0276	0.0002	0.0001	0.0000	0.0005	0.0000	0.0000	0.0001	0.0004	0.0002	0.0001	0.00227
P_Proteobacteria	C_Gammaproteobacteri	O_Betaproteobacteriales	F_Burkholderiaceae	G_Ideonella	0.0127	0.0141	0.0036	0.0000	0.0006	0.0000	0.0000	0.0000	0.0000	0.0000	0.0000	0.0001	0.0006	0.0000	0.00226
P_Verrucomicrobia	C_Verrucomicrobiae	O_Verrucomicrobiales	F_Verrucomicrobiaceae	G_Prosthecobacter	0.0000	0.0000	0.0010	0.0000	0.0165	0.0001	0.0007	0.0023	0.0003	0.0001	0.0012	0.0018	0.0070	0.0006	0.00226
P_Proteobacteria	C_Gammaproteobacteri	O_Enterobacteriales	F_Enterobacteriaceae	G_Pantoea	0.0000	0.0000	0.0000	0.0000	0.0131	0.0086	0.0003	0.0001	0.0000	0.0014	0.0046	0.0000	0.0001	0.0008	0.00208
P_Verrucomicrobia	C_Verrucomicrobiae	O_Verrucomicrobiales	F_Verrucomicrobiaceae	G_uncultured	0.0000	0.0000	0.0053	0.0003	0.0155	0.0001	0.0005	0.0020	0.0004	0.0001	0.0009	0.0021	0.0018	0.0003	0.00208
P_Actinobacteria	C_Actinobacteria	O_Frankiales	F_Geodermatophilacea	G_Blastococcus	0.0039	0.0038	0.0006	0.0196	0.0000	0.0000	0.0000	0.0000	0.0000	0.0000	0.0000	0.0001	0.0006	0.0000	0.00204
P_Proteobacteria	C_Alphaproteobacteria	O_Rhodobacterales	F_Rhodobacteraceae	G_Paracoccus	0.0098	0.0116	0.0012	0.0020	0.0001	0.0001	0.0000	0.0022	0.0000	0.0000	0.0001	0.0000	0.0000	0.0001	0.00194
P_Proteobacteria	C_Gammaproteobacteri	O_Enterobacteriales	F_Enterobacteriaceae	G_Rosenbergiella	0.0000	0.0000	0.0000	0.0000	0.0000	0.0000	0.0000	0.0000	0.0000	0.0001	0.0000	0.0000	0.0000	0.0267	0.00193
P_Proteobacteria	C_Gammaproteobacteri	O_Betaproteobacteriales	F_Burkholderiaceae	G_Massilia	0.0080	0.0105	0.0000	0.0008	0.0044	0.0000	0.0001	0.0010	0.0000	0.0000	0.0006	0.0000	0.0002	0.0000	0.00183
P_Chloroflexi	C_Chloroflexia	O_Chloroflexales	F_Chloroflexaceae	G_Chloronema	0.0002	0.0002	0.0000	0.0239	0.0000	0.0000	0.0000	0.0000	0.0000	0.0000	0.0000	0.0000	0.0000	0.0000	0.00174
P_Planctomycetes	C_Planctomycetacia	O_Gemmatales	F_Gemmataceae	G_uncultured	0.0000	0.0001	0.0104	0.0110	0.0000	0.0000	0.0000	0.0000	0.0000	0.0000	0.0001	0.0002	0.0000	0.0003	0.00159
P_Planctomycetes	C_Planctomycetacia	O_Planctomycetales	F_uncultured	G_uncultured_bacterium	0.0105	0.0100	0.0000	0.0000	0.0000	0.0000	0.0000	0.0000	0.0000	0.0000	0.0000	0.0003	0.0011	0.0001	0.00159
P_Cyanobacteria	C_Oxyphotobacteria	O_Oxyphotobacteria_Ince	F_Unknown_Family	G_Phormidium_SAG_37	0.0153	0.0065	0.0000	0.0000	0.0000	0.0000	0.0000	0.0000	0.0000	0.0000	0.0000	0.0000	0.0000	0.0000	0.00156
P_Bacteroidetes	C_Bacteroidia	O_Cytophagales	F_Spirosomaceae	G_Runella	0.0005	0.0008	0.0188	0.0000	0.0000	0.0000	0.0000	0.0000	0.0000	0.0000	0.0000	0.0000	0.0000	0.0000	0.00144
P_Firmicutes	C_Bacilli	O_Bacillales	F_Planococcaceae	G_uncultured	0.0000	0.0000	0.0195	0.0000	0.0000	0.0000	0.0000	0.0000	0.0000	0.0000	0.0000	0.0000	0.0000	0.0000	0.00139
P_Proteobacteria	C_Alphaproteobacteria	O_Rhizobiales	F_Hyphomicrobiaceae	G_Hyphomicrobium	0.0001	0.0002	0.0037	0.0131	0.0013	0.0001	0.0000	0.0000	0.0001	0.0000	0.0000	0.0002	0.0005	0.0000	0.00138
P_Actinobacteria	C_Actinobacteria	O_Micrococcales	F_Microbacteriaceae	G_SV1-3	0.0001	0.0000	0.0005	0.0009	0.0026	0.0000	0.0004	0.0013	0.0004	0.0001	0.0018	0.0049	0.0034	0.0012	0.00126
P_Firmicutes	C_Bacilli	O_Lactobacillales	F_Aerococcaceae	G_uncultured	0.0001	0.0000	0.0000	0.0000	0.0000	0.0000	0.0000	0.0000	0.0000	0.0000	0.0000	0.0001	0.0173	0.0000	0.00126
P_Actinobacteria	C_Actinobacteria	O_Corynebacteriales	F_Corynebacteriaceae	G_Corynebacterium_1	0.0006	0.0007	0.0007	0.0008	0.0011	0.0003	0.0000	0.0000	0.0003	0.0000	0.0006	0.0102	0.0005	0.0016	0.00124
P_Proteobacteria	C_Alphaproteobacteria	O_Caulobacterales	F_Caulobacteraceae	Ambiguous_taxa	0.0071	0.0080	0.0015	0.0001	0.0004	0.0000	0.0000	0.0000	0.0000	0.0000	0.0000	0.0000	0.0000	0.0000	0.00123
P_Chloroflexi	C_Chloroflexia	O_Thermomicrobiales	F_JG30-KF-CM45	G_uncultured_bacterium	0.0036	0.0036	0.0069	0.0022	0.0000	0.0000	0.0000	0.0000	0.0000	0.0001	0.0000	0.0000	0.0005	0.0002	0.00122

P_Proteobacteria	C_Alphaproteobacteria	O_Acetobacterales	F_Acetobacteraceae	G_Craurococcus	0.0072	0.0084	0.0000	0.0008	0.0000	0.0000	0.0000	0.0001	0.0000	0.0000	0.0000	0.0000	0.0000	0.0000	0.00117
P_Actinobacteria	C_Thermoleophilia	O_Solirubrobacterales	F_67-14	G_uncultured_bacterium	0.0001	0.0002	0.0152	0.0006	0.0000	0.0000	0.0000	0.0000	0.0000	0.0000	0.0000	0.0000	0.0000	0.0000	0.00115
P_Cyanobacteria	C_Oxyphotobacteria	O_Nostocales	F_Chroococcidiopsacea	G_Chroococcidiopsis_SA	0.0073	0.0085	0.0000	0.0000	0.0000	0.0000	0.0000	0.0000	0.0000	0.0000	0.0000	0.0000	0.0000	0.0000	0.00113
P_Actinobacteria	C_Actinobacteria	O_Micromonosporales	F_Micromonosporaceae	G_Actinoplanes	0.0078	0.0078	0.0002	0.0000	0.0000	0.0000	0.0000	0.0000	0.0000	0.0000	0.0000	0.0000	0.0000	0.0000	0.00112
P_Firmicutes	C_Bacilli	O_Lactobacillales	F_Streptococcaceae	G_Lactococcus	0.0000	0.0000	0.0001	0.0000	0.0000	0.0000	0.0000	0.0118	0.0000	0.0003	0.0000	0.0026	0.0000	0.0005	0.00110
P_Bacteroidetes	C_Bacteroidia	O_Sphingobacteriales	F_NS11-12_marine_gro	G_uncultured_bacterium	0.0000	0.0000	0.0000	0.0000	0.0102	0.0000	0.0001	0.0022	0.0000	0.0000	0.0002	0.0002	0.0020	0.0000	0.00107
P_Planctomycetes	C_Planctomycetacia	O_Isosphaerales	F_Isosphaeraceae	G_uncultured	0.0011	0.0010	0.0060	0.0067	0.0000	0.0000	0.0000	0.0000	0.0000	0.0000	0.0000	0.0000	0.0000	0.0000	0.00106
P_Proteobacteria	C_Alphaproteobacteria	O_Sphingomonadales	F_Sphingomonadaceae	G_uncultured	0.0003	0.0002	0.0088	0.0033	0.0008	0.0000	0.0000	0.0004	0.0000	0.0000	0.0001	0.0002	0.0004	0.0001	0.00105
P_Proteobacteria	C_Alphaproteobacteria	O_Sphingomonadales	F_Sphingomonadaceae	G_Novosphingobium	0.0044	0.0049	0.0027	0.0020	0.0000	0.0000	0.0000	0.0002	0.0000	0.0000	0.0001	0.0001	0.0001	0.0000	0.00103
P_Actinobacteria	C_Acidimicrobiia	O_Microtrichales	F_Iamiaceae	G_Iamia	0.0008	0.0012	0.0032	0.0065	0.0000	0.0000	0.0003	0.0001	0.0001	0.0000	0.0003	0.0002	0.0013	0.0001	0.00101
P_Proteobacteria	C_Alphaproteobacteria	O_Sphingomonadales	F_Sphingomonadaceae	G_Sphingopyxis	0.0048	0.0068	0.0014	0.0001	0.0001	0.0000	0.0000	0.0003	0.0000	0.0000	0.0000	0.0000	0.0007	0.0000	0.00101
P_Planctomycetes	C_Planctomycetacia	O_Pirellulales	F_Pirellulaceae	G_Pir4_lineage	0.0046	0.0041	0.0025	0.0027	0.0000	0.0000	0.0000	0.0000	0.0000	0.0000	0.0000	0.0000	0.0000	0.0000	0.00099
P_Actinobacteria	C_Actinobacteria	O_Micrococcales	F_Intrasporangiaceae	G_Janibacter	0.0004	0.0003	0.0030	0.0090	0.0000	0.0000	0.0001	0.0000	0.0001	0.0000	0.0001	0.0007	0.0000	0.0001	0.00099
P_Bacteroidetes	C_Bacteroidia	O_Flavobacteriales	F_Crocinitomicaceae	G_Fluviicola	0.0001	0.0001	0.0078	0.0005	0.0024	0.0000	0.0000	0.0009	0.0002	0.0000	0.0004	0.0007	0.0005	0.0001	0.00097
P_Firmicutes	C_Bacilli	O_Lactobacillales	F_Leuconostocaceae	G_Leuconostoc	0.0000	0.0000	0.0003	0.0000	0.0000	0.0000	0.0000	0.0000	0.0001	0.0000	0.0000	0.0088	0.0000	0.0042	0.00095
P_Proteobacteria	C_Gammaproteobacteri	O_Betaproteobacteriales	F_Burkholderiaceae	G_Aquabacterium	0.0005	0.0007	0.0087	0.0010	0.0019	0.0000	0.0000	0.0000	0.0000	0.0000	0.0000	0.0000	0.0003	0.0000	0.00093
P_Actinobacteria	C_Actinobacteria	O_Frankiales	F_Geodermatophilacea	G_Geodermatophilus	0.0026	0.0026	0.0005	0.0026	0.0042	0.0000	0.0000	0.0000	0.0004	0.0000	0.0000	0.0000	0.0000	0.0000	0.00092
P_Chloroflexi	C_Chloroflexia	O_Kallotenuales	F_AKIW781	G_uncultured_bacterium	0.0056	0.0069	0.0000	0.0000	0.0000	0.0000	0.0000	0.0000	0.0000	0.0000	0.0000	0.0000	0.0000	0.0001	0.00090
P_Proteobacteria	C_Alphaproteobacteria	O_Paracaedibacterales	F_Paracaedibacteracea	G_uncultured	0.0000	0.0000	0.0050	0.0043	0.0030	0.0000	0.0000	0.0000	0.0000	0.0000	0.0000	0.0000	0.0001	0.0000	0.00089
P_Proteobacteria	C_Gammaproteobacteri	O_Betaproteobacteriales	F_Burkholderiaceae	G_Comamonas	0.0004	0.0003	0.0075	0.0001	0.0020	0.0000	0.0001	0.0001	0.0000	0.0004	0.0001	0.0004	0.0004	0.0002	0.00086
P_Firmicutes	C_Bacilli	O_Bacillales	F_Alicyclobacillaceae	G_Tumebacillus	0.0000	0.0000	0.0000	0.0000	0.0000	0.0000	0.0000	0.0000	0.0000	0.0000	0.0000	0.0000	0.0117	0.0000	0.00084
P_Actinobacteria	C_Actinobacteria	O_Micrococcales	F_Brevibacteriaceae	G_Brevibacterium	0.0003	0.0001	0.0007	0.0102	0.0000	0.0000	0.0000	0.0000	0.0000	0.0000	0.0000	0.0000	0.0000	0.0000	0.00081
P_Bacteroidetes	C_Bacteroidia	O_Cytophagales	F_Spirosomaceae	G_Emticicia	0.0000	0.0000	0.0110	0.0000	0.0000	0.0000	0.0000	0.0000	0.0000	0.0000	0.0000	0.0000	0.0000	0.0000	0.00079
P_Planctomycetes	C_Planctomycetacia	O_Gemmatales	F_Gemmataceae	G_Gemmata	0.0005	0.0005	0.0027	0.0013	0.0042	0.0000	0.0003	0.0000	0.0000	0.0000	0.0001	0.0003	0.0009	0.0000	0.00078
P_Cyanobacteria	C_Oxyphotobacteria	O_Nostocales	F_Chroococcidiopsacea	G_uncultured	0.0048	0.0057	0.0000	0.0000	0.0000	0.0000	0.0000	0.0000	0.0000	0.0000	0.0000	0.0000	0.0000	0.0000	0.00075
P_Chloroflexi	C_Chloroflexia	O_Chloroflexales	F_Herpetosiphonaceae	G_Herpetosiphon	0.0050	0.0052	0.0000	0.0000	0.0000	0.0000	0.0000	0.0000	0.0000	0.0000	0.0000	0.0000	0.0000	0.0000	0.00073
P_Bacteroidetes	C_Bacteroidia	O_Chitinophagales	F_Saprospiraceae	G_uncultured	0.0000	0.0000	0.0028	0.0002	0.0045	0.0000	0.0001	0.0011	0.0000	0.0000	0.0002	0.0003	0.0006	0.0002	0.00072
P_Proteobacteria	C_Alphaproteobacteria	O_Sphingomonadales	F_Sphingomonadaceae	Ambiguous_taxa	0.0008	0.0008	0.0009	0.0016	0.0044	0.0000	0.0000	0.0009	0.0000	0.0000	0.0000	0.0000	0.0004	0.0000	0.00071
P_Proteobacteria	C_Gammaproteobacteri	O_Betaproteobacteriales	F_Leeiaceae	G_Leeia	0.0000	0.0000	0.0099	0.0000	0.0000	0.0000	0.0000	0.0000	0.0000	0.0000	0.0000	0.0000	0.0000	0.0000	0.00071
P_Planctomycetes	C_Planctomycetacia	O_Isosphaerales	F_Isosphaeraceae	G_Singulisphaera	0.0005	0.0004	0.0003	0.0085	0.0000	0.0000	0.0000	0.0000	0.0000	0.0000	0.0000	0.0000	0.0000	0.0000	0.00070
P_Planctomycetes	C_Planctomycetacia	O_Planctomycetales	F_Rubinisphaeraceae	G_SH-PL14	0.0005	0.0006	0.0008	0.0005	0.0016	0.0001	0.0000	0.0025	0.0001	0.0000	0.0004	0.0005	0.0017	0.0002	0.00068
P_Bacteroidetes	C_Bacteroidia	O_Chitinophagales	F_Chitinophagaceae	G_Flavihumibacter	0.0038	0.0048	0.0000	0.0000	0.0000	0.0000	0.0000	0.0000	0.0000	0.0000	0.0000	0.0000	0.0009	0.0000	0.00068
P_Actinobacteria	C_Rubrobacteria	O_Rubrobacterales	F_Rubrobacteriaceae	G_Rubrobacter	0.0018	0.0025	0.0052	0.0000	0.0000	0.0000	0.0000	0.0000	0.0000	0.0000	0.0000	0.0000	0.0000	0.0000	0.00068
P_Chlamydiae	C_Chlamydiae	O_Chlamydiales	F_Parachlamydiaceae	G_Candidatus_Protochla	0.0024	0.0037	0.0002	0.0030	0.0000	0.0000	0.0000	0.0000	0.0000	0.0000	0.0000	0.0000	0.0000	0.0000	0.00067
P_Proteobacteria	C_Gammaproteobacteri	O_Betaproteobacteriales	F_Burkholderiaceae	G_Caenimonas	0.0000	0.0001	0.0001	0.0001	0.0090	0.0000	0.0000	0.0000	0.0000	0.0000	0.0001	0.0000	0.0000	0.0000	0.00066
P_Chloroflexi	C_Chloroflexia	O_Chloroflexales	F_Chloroflexaceae	G_Chloroflexus	0.0000	0.0000	0.0000	0.0087	0.0000	0.0000	0.0000	0.0000	0.0000	0.0000	0.0000	0.0000	0.0000	0.0000	0.00062
P_Planctomycetes	C_Planctomycetacia	O_Pirellulales	F_Pirellulaceae	G_Pirellula	0.0009	0.0009	0.0004	0.0000	0.0043	0.0000	0.0000	0.0000	0.0001	0.0000	0.0002	0.0006	0.0012	0.0000	0.00062
P_Proteobacteria	C_Alphaproteobacteria	O_Rhizobiales	F_Xanthobacteraceae	G_Bradyrhizobium	0.0004	0.0004	0.0011	0.0056	0.0011	0.0000	0.0000	0.0000	0.0000	0.0000	0.0000	0.0000	0.0000	0.0000	0.00062
P_Patescibacteria	C_Saccharimonadia	O_Saccharimonadales	F_uncultured_bacterium	G_uncultured_bacterium	0.0014	0.0016	0.0008	0.0011	0.0000	0.0001	0.0002	0.0009	0.0001	0.0000	0.0001	0.0007	0.0014	0.0000	0.00060
P_Firmicutes	C_Clostridia	O_Clostridiales	F_Clostridiaceae_1	G_Clostridium_sensu_st	0.0001	0.0002	0.0002	0.0000	0.0050	0.0000	0.0000	0.0008	0.0000	0.0000	0.0000	0.0012	0.0000	0.0009	0.00060
P_Proteobacteria	C_Gammaproteobacteri	O_Betaproteobacteriales	F_Chromobacteriaceae	G_Vogesella	0.0000	0.0001	0.0082	0.0000	0.0000	0.0000	0.0000	0.0000	0.0000	0.0000	0.0000	0.0000	0.0000	0.0000	0.00059
P_Firmicutes	C_Clostridia	O_Clostridiales	F_Peptostreptococcacea	G_uncultured	0.0001	0.0001	0.0007	0.0034	0.0035	0.0000	0.0000	0.0000	0.0001	0.0000	0.0000	0.0003	0.0000	0.0000	0.00059
P_Planctomycetes	C_Planctomycetacia	O_Gemmatales	F_Gemmataceae	G_Fimbriiglobus	0.0001	0.0002	0.0019	0.0000	0.0030	0.0000	0.0006	0.0002	0.0003	0.0000	0.0004	0.0005	0.0007	0.0004	0.00059
P_Proteobacteria	C_Alphaproteobacteria	O_Rhodobacterales	F_Rhodobacteraceae	G_Rubellimicrobium	0.0017	0.0021	0.0001	0.0005	0.0035	0.0000	0.0000	0.0000	0.0000	0.0000	0.0000	0.0000	0.0000	0.0000	0.00057
P_Deinococcus-The	C_Deinococci	O_Deinococcales	F_Deinococcaceae	G_Deinococcus	0.0024	0.0024	0.0001	0.0028	0.0000	0.0000	0.0000	0.0000	0.0001	0.0000	0.0000	0.0000	0.0000	0.0001	0.00057
P_Actinobacteria	C_Actinobacteria	O_Frankiales	F_Geodermatophilacea	Ambiguous_taxa	0.0000	0.0001	0.0005	0.0073	0.0000	0.0000	0.0000	0.0000	0.0000	0.0000	0.0000	0.0000	0.0000	0.0000	0.00056
P_Firmicutes	C_Clostridia	O_Clostridiales	F_Clostridiaceae_1	G_Clostridium_sensu_st	0.0000	0.0000	0.0079	0.0000	0.0000	0.0000	0.0000	0.0000	0.0000	0.0000	0.0000	0.0000	0.0000	0.0000	0.00056
P_Proteobacteria	C_Gammaproteobacteri	O_Betaproteobacteriales	F_Burkholderiaceae	G_Ramlibacter	0.0017	0.0021	0.0005	0.0000	0.0032	0.0000	0.0000	0.0002	0.0000	0.0000	0.0000	0.0001	0.0000	0.0000	0.00056
P_Proteobacteria	C_Gammaproteobacteri	O_Cellvibrionales	F_Cellvibrionaceae	G_uncultured	0.0005	0.0004	0.0069	0.0000	0.0000	0.0000	0.0000	0.0000	0.0000	0.0000	0.0000	0.0000	0.0000	0.0000	0.00055
P_Proteobacteria	C_Gammaproteobacteri	O_Enterobacteriales	F_Enterobacteriaceae	G_Escherichia-Shigella	0.0000	0.0000	0.0000	0.0000	0.0007	0.0000	0.0000	0.0000	0.0058	0.0010	0.0000	0.0000	0.0000	0.0000	0.00054
P_Patescibacteria	C_Gracilibacteria	O_Candidatus_Peribacter	F_uncultured_bacterium	G_uncultured_bacterium	0.0000	0.0000	0.0001	0.0000	0.0050	0.0000	0.0000	0.0010	0.0000	0.0000	0.0001	0.0001	0.0012	0.0001	0.00054
P_Bacteroidetes	C_Bacteroidia	O_Chitinophagales	F_Chitinophagaceae	G_uncultured	0.0019	0.0018	0.0008	0.0000	0.0005	0.0000	0.0001	0.0011	0.0000	0.0000	0.0002	0.0001	0.0006	0.0001	0.00052
P_Patescibacteria	C_Saccharimonadia	O_Saccharimonadales	F_Saccharimonadaceae	G_uncultured_bacterium	0.0002	0.0003	0.0033	0.0018	0.0008	0.0000	0.0000	0.0009	0.0000	0.0000	0.0000	0.0000	0.0000	0.0000	0.00052
P_Bacteroidetes	C_Bacteroidia	O_Flavobacteriales	F_Weeksellaceae	G_Chishuiella	0.0000	0.0000	0.0001	0.0000	0.0000	0.0000	0.0000	0.0000	0.0000	0.0003	0.0068	0.0000	0.0000	0.0000	0.00052
P_Actinobacteria	C_Actinobacteria	O_Propionibacteriales	F_Nocardioidaceae	G_Aeromicrobium	0.0015	0.0014	0.0042	0.0000	0.0000	0.0000	0.0000	0.0001	0.0000	0.0000	0.0000	0.0000	0.0000	0.0000	0.00051
P_Firmicutes	C_Bacilli	O_Lactobacillales	F_Streptococcaceae	G_Streptococcus	0.0001	0.0001	0.0011	0.0003	0.0021	0.0001	0.0000	0.0026	0.0003	0.0001	0.0003	0.0002	0.0000	0.0001	0.00051
P_Actinobacteria	C_Actinobacteria	O_Micrococcales	F_Dermabacteraceae	G_Brachybacterium	0.0009	0.0011	0.0002	0.0024	0.0009	0.0000	0.0000	0.0000	0.0003	0.0000	0.0000	0.0009	0.0000	0.0004	0.00051
P_Proteobacteria	C_Alphaproteobacteria	O_Rhizobiales	F_Xanthobacteraceae	G_Pseudorhodoplanes	0.0007	0.0005	0.0010	0.0049	0.0000	0.0000	0.0000	0.0000	0.0000	0.0000	0.0000	0.0000	0.0000	0.0000	0.00051
P_Proteobacteria	C_Gammaproteobacteri	O_Xanthomonadales	F_Xanthomonadaceae	G_Luteimonas	0.0036	0.0027	0.0006	0.0001	0.0000	0.0000	0.0000	0.0000	0.0000	0.0000	0.0000	0.0000	0.0000	0.0000	0.00050
P_Proteobacteria	C_Gammaproteobacteri	O_Enterobacteriales	F_Enterobacteriaceae	G_Serratia	0.0000	0.0000	0.0000	0.0000	0.0000	0.0000	0.0067	0.0000	0.0000	0.0000	0.0001	0.0001	0.0000	0.0000	0.00049
P_Proteobacteria	C_Gammaproteobacteri	O_Xanthomonadales	F_Rhodanobacteraceae	G_Aquimonas	0.0030	0.0038	0.0001	0.0000	0.0000	0.0000	0.0000	0.0000	0.0000	0.0000	0.0000	0.0000	0.0000	0.0000	0.00049
P_Firmicutes	C_Clostridia	O_Clostridiales	F_Ruminococcaceae	G_Anaerobacterium	0.0000	0.0000	0.0065	0.0000	0.0000	0.0000	0.0000	0.0000	0.0000	0.0000	0.0000	0.0000	0.0000	0.0000	0.00046
P_Actinobacteria	C_Actinobacteria	O_Corynebacteriales	F_Nocardiaceae	G_Nocardia	0.0015	0.0014	0.0029	0.0007	0.0000	0.0000	0.0000	0.0000	0.0000	0.0000	0.0000	0.0000	0.0000	0.0000	0.00046
P_Firmicutes	C_Bacilli	O_Lactobacillales	F_Enterococcaceae	G_Enterococcus	0.0001	0.0002	0.0000	0.0001	0.0000	0.0000	0.0000	0.0000	0.0000	0.0046	0.0000	0.0002	0.0000	0.0013	0.00046
P_Proteobacteria	C_Alphaproteobacteria	O_Rhizobiales	F_Rhizobiaceae	Ambiguous_taxa	0.0033	0.0026	0.0004	0.0000	0.0000	0.0000	0.0000	0.0000	0.0000	0.0000	0.0000	0.0000	0.0000	0.0000	0.00045
P_Proteobacteria	C_Alphaproteobacteria	O_Micavibrionales	F_uncultured	G_uncultured_bacterium	0.0006	0.0004	0.0043	0.0006	0.0003	0.0000	0.0000	0.0000	0.0000	0.0000	0.0000	0.0000	0.0000	0.0000	0.00044
P_Proteobacteria	C_Alphaproteobacteria	O_Tistrellales	F_Geminicoccaceae	G_Candidatus_Alysiosph	0.0007	0.0007	0.0041	0.0006	0.0000	0.0000	0.0000	0.0000	0.0000	0.0000	0.0000	0.0000	0.0000	0.0000	0.00044
P_Proteobacteria	C_Alphaproteobacteria	O_Rhizobiales	F_Stappiaceae	Ambiguous_taxa	0.0008	0.0007	0.0044	0.0001	0.0000	0.0000	0.0000	0.0000	0.0000	0.0000	0.0000	0.0000	0.0000	0.0000	0.00043
P_Gemmatimonade	C_Gemmatimonadetes	O_Gemmatimonadales	F_Gemmatimonadaceae	G_Gemmatirosa	0.0028	0.0031	0.0000	0.0000	0.0000	0.0000	0.0000	0.0000	0.0000	0.0000	0.0000	0.0000	0.0000	0.0000	0.00042

P_Proteobacteria	C_Alphaproteobacteria	O_Caulobacterales	F_Caulobacteraceae	G_Phenylobacterium	0.0022	0.0027	0.0005	0.0003	0.0000	0.0000	0.0000	0.0000	0.0000	0.0000	0.0000	0.0001	0.0000	0.0000	0.00041
P_Proteobacteria	C_Alphaproteobacteria	O_Sphingomonadales	F_Sphingomonadaceae	G_Zymomonas	0.0000	0.0000	0.0000	0.0000	0.0000	0.0000	0.0000	0.0000	0.0000	0.0000	0.0000	0.0044	0.0000	0.0013	0.00041
P_Bacteroidetes	C_Bacteroidia	O_Chitinophagales	F_uncultured	G_metagenome	0.0000	0.0000	0.0001	0.0000	0.0030	0.0000	0.0002	0.0018	0.0000	0.0000	0.0000	0.0001	0.0005	0.0001	0.00041
P_Acidobacteria	C_Blastocatellia_(Subgro	O_Blastocatellales	F_Blastocatellaceae	Ambiguous_taxa	0.0027	0.0030	0.0000	0.0000	0.0000	0.0000	0.0000	0.0000	0.0000	0.0000	0.0000	0.0000	0.0000	0.0000	0.00041
P_Proteobacteria	C_Alphaproteobacteria	O_Sphingomonadales	F_Sphingomonadaceae	G_Porphyrobacter	0.0011	0.0014	0.0003	0.0002	0.0024	0.0000	0.0000	0.0001	0.0000	0.0000	0.0000	0.0000	0.0000	0.0000	0.00041
P_Proteobacteria	C_Alphaproteobacteria	O_Rhizobiales	F_Beijerinckiaceae	G_Microvirga	0.0015	0.0013	0.0008	0.0020	0.0000	0.0000	0.0000	0.0000	0.0000	0.0000	0.0000	0.0000	0.0000	0.0000	0.00040
P_Actinobacteria	C_Actinobacteria	O_Micromonosporales	F_Micromonosporaceae	G_Micromonospora	0.0027	0.0029	0.0000	0.0000	0.0000	0.0000	0.0000	0.0000	0.0000	0.0000	0.0000	0.0000	0.0000	0.0000	0.00040
P_Actinobacteria	C_Actinobacteria	O_Micrococcales	F_Microbacteriaceae	Ambiguous_taxa	0.0016	0.0016	0.0007	0.0007	0.0000	0.0000	0.0001	0.0000	0.0000	0.0001	0.0001	0.0006	0.0001	0.0001	0.00040
P_Proteobacteria	C_Alphaproteobacteria	O_Caulobacterales	F_Caulobacteraceae	G_Caulobacter	0.0021	0.0022	0.0009	0.0001	0.0000	0.0000	0.0001	0.0000	0.0000	0.0000	0.0000	0.0001	0.0000	0.0000	0.00040
P_Proteobacteria	C_Gammaproteobacteria	O_Betaproteobacteriales	F_Burkholderiaceae	G_uncultured	0.0009	0.0008	0.0017	0.0001	0.0001	0.0000	0.0000	0.0013	0.0000	0.0000	0.0001	0.0001	0.0002	0.0000	0.00038
P_Bacteroidetes	C_Bacteroidia	O_Sphingobacteriales	F_NS11-12_marine_gro	Ambiguous_taxa	0.0000	0.0000	0.0004	0.0002	0.0008	0.0001	0.0000	0.0002	0.0001	0.0000	0.0001	0.0004	0.0028	0.0001	0.00037
P_Dependentiae	C_Babeliae	O_Babeliales	F_uncultured_bacterium	G_uncultured_bacterium	0.0000	0.0001	0.0001	0.0010	0.0019	0.0000	0.0000	0.0011	0.0000	0.0000	0.0000	0.0001	0.0007	0.0000	0.00037
P_Actinobacteria	C_Actinobacteria	O_Streptomycetales	F_Streptomycetaceae	G_Streptomyces	0.0021	0.0027	0.0003	0.0000	0.0000	0.0000	0.0000	0.0000	0.0000	0.0000	0.0000	0.0000	0.0000	0.0000	0.00037
P_Cyanobacteria	C_Oxyphotobacteria	O_Nostocales	F_uncultured	Ambiguous_taxa	0.0021	0.0029	0.0000	0.0000	0.0000	0.0000	0.0000	0.0000	0.0000	0.0000	0.0000	0.0000	0.0000	0.0000	0.00036
P_Actinobacteria	C_Actinobacteria	O_Micrococcales	F_Microbacteriaceae	G_Microbacterium	0.0010	0.0011	0.0002	0.0021	0.0001	0.0000	0.0000	0.0000	0.0000	0.0000	0.0001	0.0001	0.0001	0.0001	0.00036
P_Proteobacteria	C_Deltaproteobacteria	O_Bdellovibrionales	F_Bdellovibrionaceae	G_Bdellovibrio	0.0012	0.0014	0.0012	0.0000	0.0000	0.0000	0.0003	0.0000	0.0001	0.0000	0.0002	0.0002	0.0000	0.0003	0.00036
P_Acidobacteria	C_Acidobacteriia	O_Solibacterales	F_Solibacteraceae_(Sub	G_Bryobacter	0.0003	0.0006	0.0000	0.0000	0.0023	0.0000	0.0002	0.0006	0.0001	0.0000	0.0001	0.0005	0.0000	0.0002	0.00035
P_Proteobacteria	C_Alphaproteobacteria	O_Rhizobiales	F_Rhizobiaceae	G_Mesorhizobium	0.0015	0.0014	0.0014	0.0006	0.0000	0.0000	0.0000	0.0000	0.0000	0.0000	0.0000	0.0000	0.0000	0.0000	0.00035
P_Firmicutes	C_Bacilli	O_Lactobacillales	F_Lactobacillaceae	G_Lactobacillus	0.0001	0.0002	0.0002	0.0004	0.0000	0.0000	0.0000	0.0022	0.0000	0.0000	0.0003	0.0007	0.0000	0.0006	0.00035
P_Proteobacteria	C_Gammaproteobacteria	O_Xanthomonadales	F_Xanthomonadaceae	G_Lysobacter	0.0008	0.0011	0.0007	0.0000	0.0010	0.0000	0.0000	0.0006	0.0000	0.0000	0.0001	0.0003	0.0002	0.0000	0.00035
P_Proteobacteria	C_Alphaproteobacteria	O_Azospirillales	F_Azospirillaceae	G_Azospirillum	0.0021	0.0026	0.0000	0.0000	0.0000	0.0000	0.0000	0.0000	0.0000	0.0000	0.0000	0.0000	0.0000	0.0000	0.00034
P_Bacteroidetes	C_Bacteroidia	O_Flavobacteriales	F_Weeksellaceae	G_Moheibacter	0.0020	0.0027	0.0000	0.0000	0.0000	0.0000	0.0000	0.0000	0.0000	0.0000	0.0000	0.0000	0.0000	0.0000	0.00034
P_Bacteroidetes	C_Bacteroidia	O_Cytophagales	F_Spirosomaceae	G_Pseudarcicella	0.0000	0.0000	0.0001	0.0000	0.0006	0.0000	0.0000	0.0029	0.0000	0.0000	0.0001	0.0003	0.0003	0.0001	0.00032
P_Proteobacteria	C_Alphaproteobacteria	O_Rhizobiales	F_Rhizobiales_Incertae	G_uncultured	0.0001	0.0000	0.0001	0.0000	0.0036	0.0000	0.0000	0.0000	0.0000	0.0000	0.0000	0.0002	0.0004	0.0000	0.00032
P_Proteobacteria	C_Alphaproteobacteria	O_Sphingomonadales	F_Sphingomonadaceae	G_Altererythrobacter	0.0004	0.0005	0.0001	0.0002	0.0027	0.0000	0.0000	0.0000	0.0000	0.0000	0.0000	0.0001	0.0004	0.0000	0.00031
P_Firmicutes	C_Clostridia	O_Clostridiales	F_Clostridiaceae_1	G_Clostridium_sensu_st	0.0000	0.0000	0.0008	0.0023	0.0007	0.0000	0.0001	0.0000	0.0000	0.0000	0.0002	0.0000	0.0000	0.0003	0.00031
P_Deinococcus-The	C_Deinococci	O_Deinococcales	F_Trueperaceae	G_Truepera	0.0021	0.0021	0.0001	0.0000	0.0000	0.0000	0.0000	0.0000	0.0000	0.0000	0.0000	0.0000	0.0000	0.0000	0.00031
P_Chloroflexi	C_Chloroflexia	O_Chloroflexales	F_Chloroflexaceae	G_Candidatus_Chloropl	0.0000	0.0000	0.0000	0.0043	0.0000	0.0000	0.0000	0.0000	0.0000	0.0000	0.0000	0.0000	0.0000	0.0000	0.00031
P_Chloroflexi	C_Chloroflexia	O_Chloroflexales	F_Roseiflexaceae	G_uncultured	0.0012	0.0014	0.0001	0.0000	0.0009	0.0000	0.0001	0.0000	0.0001	0.0000	0.0000	0.0000	0.0005	0.0000	0.00031
P_Actinobacteria	C_Acidimicrobiia	O_Microtrichales	F_uncultured	G_uncultured_bacterium	0.0005	0.0008	0.0003	0.0023	0.0000	0.0000	0.0000	0.0000	0.0000	0.0000	0.0000	0.0001	0.0003	0.0000	0.00031
P_Proteobacteria	C_Alphaproteobacteria	O_Rhodobacterales	F_Rhodobacteraceae	G_Amaricoccus	0.0000	0.0000	0.0004	0.0038	0.0000	0.0000	0.0000	0.0000	0.0000	0.0000	0.0000	0.0000	0.0000	0.0000	0.00030
P_Proteobacteria	C_Alphaproteobacteria	O_Acetobacterales	F_Acetobacteraceae	G_Acetobacter	0.0000	0.0000	0.0000	0.0000	0.0042	0.0000	0.0000	0.0000	0.0000	0.0000	0.0000	0.0000	0.0000	0.0000	0.00030
P_Actinobacteria	C_Actinobacteria	O_Frankiales	F_Sporichthyaceae	G_hgcI_clade	0.0000	0.0000	0.0001	0.0001	0.0013	0.0000	0.0000	0.0000	0.0000	0.0000	0.0000	0.0004	0.0022	0.0000	0.00030
P_Proteobacteria	C_Alphaproteobacteria	O_Rhizobiales	F_Beijerinckiaceae	G_Bosea	0.0008	0.0009	0.0005	0.0004	0.0013	0.0000	0.0002	0.0000	0.0001	0.0000	0.0000	0.0000	0.0000	0.0000	0.00030
P_Actinobacteria	C_Actinobacteria	O_Actinomycetales	F_Actinomycetaceae	G_uncultured	0.0000	0.0000	0.0000	0.0000	0.0000	0.0000	0.0000	0.0042	0.0000	0.0000	0.0000	0.0000	0.0000	0.0000	0.00030
P_Planctomycetes	C_Planctomycetacia	O_Pirellulales	F_Pirellulaceae	G_uncultured	0.0000	0.0001	0.0025	0.0000	0.0000	0.0000	0.0000	0.0000	0.0000	0.0000	0.0000	0.0005	0.0007	0.0003	0.00029
P_Firmicutes	C_Bacilli	O_Bacillales	F_Staphylococcaceae	G_Salinicoccus	0.0000	0.0000	0.0000	0.0001	0.0000	0.0011	0.0000	0.0003	0.0024	0.0000	0.0001	0.0000	0.0000	0.0001	0.00029
P_Planctomycetes	C_Planctomycetacia	O_Pirellulales	F_Pirellulaceae	G_Rhodopirellula	0.0018	0.0009	0.0004	0.0000	0.0000	0.0000	0.0000	0.0009	0.0000	0.0000	0.0000	0.0000	0.0000	0.0000	0.00028
P_Actinobacteria	C_Actinobacteria	O_Propionibacteriales	F_Propionibacteriaceae	G_Cutibacterium	0.0001	0.0003	0.0004	0.0005	0.0004	0.0001	0.0000	0.0005	0.0001	0.0000	0.0005	0.0002	0.0007	0.0001	0.00028
P_Bacteroidetes	C_Bacteroidia	O_Cytophagales	F_Spirosomaceae	G_uncultured	0.0020	0.0018	0.0000	0.0000	0.0000	0.0000	0.0000	0.0000	0.0000	0.0000	0.0000	0.0000	0.0000	0.0000	0.00028
P_Chlamydiae	C_Chlamydiae	O_Chlamydiales	F_Parachlamydiaceae	G_Neochlamydia	0.0002	0.0003	0.0002	0.0021	0.0005	0.0000	0.0000	0.0000	0.0000	0.0000	0.0000	0.0000	0.0004	0.0000	0.00028
P_Patescibacteria	C_Parcubacteria	O_Candidatus_Kaiserbac	F_uncultured_bacterium	G_uncultured_bacterium	0.0001	0.0003	0.0003	0.0000	0.0026	0.0000	0.0000	0.0000	0.0000	0.0000	0.0000	0.0001	0.0000	0.0002	0.00026
P_Bacteroidetes	C_Bacteroidia	O_Cytophagales	F_Microscillaceae	G_uncultured	0.0002	0.0004	0.0002	0.0000	0.0010	0.0000	0.0000	0.0000	0.0001	0.0000	0.0001	0.0004	0.0011	0.0002	0.00026
P_Proteobacteria	C_Gammaproteobacteria	O_Pasteurellales	F_Pasteurellaceae	G_Haemophilus	0.0000	0.0000	0.0000	0.0000	0.0031	0.0000	0.0000	0.0000	0.0000	0.0000	0.0000	0.0000	0.0004	0.0000	0.00025
P_Actinobacteria	C_Actinobacteria	O_Micrococcales	F_Cellulomonadaceae	G_Cellulomonas	0.0019	0.0013	0.0001	0.0001	0.0000	0.0000	0.0000	0.0000	0.0000	0.0000	0.0000	0.0000	0.0000	0.0001	0.00025
P_Proteobacteria	C_Alphaproteobacteria	O_Acetobacterales	F_Acetobacteraceae	G_uncultured	0.0010	0.0015	0.0001	0.0000	0.0000	0.0000	0.0000	0.0000	0.0000	0.0000	0.0000	0.0008	0.0000	0.0001	0.00025
P_Actinobacteria	C_Actinobacteria	O_Micrococcales	F_Promicromonosporac	G_Cellulosimicrobium	0.0016	0.0018	0.0001	0.0000	0.0000	0.0000	0.0000	0.0000	0.0000	0.0000	0.0000	0.0000	0.0000	0.0000	0.00025
P_Actinobacteria	C_Actinobacteria	O_Corynebacteriales	F_Corynebacteriaceae	G_Lawsonella	0.0000	0.0001	0.0003	0.0002	0.0007	0.0001	0.0000	0.0013	0.0002	0.0000	0.0001	0.0002	0.0001	0.0000	0.00025
P_Planctomycetes	C_Planctomycetacia	O_Planctomycetales	F_Schlesneriaceae	G_Schlesneria	0.0000	0.0000	0.0004	0.0000	0.0012	0.0001	0.0000	0.0000	0.0001	0.0000	0.0001	0.0007	0.0006	0.0001	0.00024
P_Bacteroidetes	C_Bacteroidia	O_Bacteroidales	F_Prolixibacteraceae	G_uncultured	0.0000	0.0000	0.0001	0.0000	0.0033	0.0000	0.0000	0.0000	0.0000	0.0000	0.0000	0.0000	0.0000	0.0000	0.00024
P_Proteobacteria	C_Alphaproteobacteria	O_Rhizobiales	F_Xanthobacteraceae	G_Ancylobacter	0.0017	0.0016	0.0000	0.0000	0.0000	0.0000	0.0000	0.0000	0.0000	0.0000	0.0000	0.0000	0.0000	0.0000	0.00024
P_Actinobacteria	C_Thermoleophilia	O_Solirubrobacterales	F_67-14	Ambiguous_taxa	0.0007	0.0007	0.0017	0.0003	0.0000	0.0000	0.0000	0.0000	0.0000	0.0000	0.0000	0.0000	0.0000	0.0000	0.00024
P_Gemmatimonade	C_Gemmatimonadetes	O_Gemmatimonadales	F_Gemmatimonadaceae	G_Gemmatimonas	0.0013	0.0014	0.0000	0.0000	0.0000	0.0000	0.0000	0.0001	0.0000	0.0000	0.0001	0.0005	0.0000	0.0000	0.00024
P_Planctomycetes	C_Planctomycetacia	O_Planctomycetales	F_Rubinisphaeraceae	G_uncultured	0.0000	0.0000	0.0004	0.0000	0.0000	0.0000	0.0000	0.0000	0.0001	0.0000	0.0001	0.0005	0.0022	0.0000	0.00024
P_Proteobacteria	C_Gammaproteobacteria	O_Alteromonadales	F_Idiomarinaceae	G_Idiomarina	0.0015	0.0018	0.0000	0.0000	0.0000	0.0000	0.0000	0.0000	0.0000	0.0000	0.0000	0.0000	0.0000	0.0000	0.00023
P_Firmicutes	C_Bacilli	O_Lactobacillales	F_Carnobacteriaceae	G_Alloiococcus	0.0000	0.0000	0.0000	0.0000	0.0031	0.0000	0.0000	0.0000	0.0000	0.0000	0.0001	0.0000	0.0000	0.0000	0.00023
P_Armatimonadetes	C_Armatimonadia	O_Armatimonadales	F_Armatimonadaceae	G_Armatimonas	0.0000	0.0000	0.0001	0.0000	0.0022	0.0000	0.0000	0.0006	0.0000	0.0000	0.0000	0.0002	0.0000	0.0001	0.00023
P_Bacteroidetes	C_Bacteroidia	O_Cytophagales	F_Cyclobacteriaceae	G_Algoriphagus	0.0014	0.0016	0.0001	0.0000	0.0000	0.0000	0.0000	0.0000	0.0000	0.0000	0.0000	0.0000	0.0000	0.0000	0.00022
P_Proteobacteria	C_Gammaproteobacteria	O_Betaproteobacteriales	F_Burkholderiaceae	G_Delftia	0.0000	0.0001	0.0030	0.0000	0.0000	0.0000	0.0000	0.0000	0.0000	0.0000	0.0000	0.0000	0.0000	0.0000	0.00022
P_Patescibacteria	C_Gracilibacteria	O_Absconditabacteriales_	Ambiguous_taxa	Ambiguous_taxa	0.0000	0.0000	0.0000	0.0000	0.0016	0.0000	0.0000	0.0005	0.0000	0.0000	0.0000	0.0000	0.0009	0.0001	0.00022
P_Proteobacteria	C_Alphaproteobacteria	O_Rhizobiales	F_Xanthobacteraceae	G_Pseudolabrys	0.0001	0.0000	0.0006	0.0024	0.0000	0.0000	0.0000	0.0000	0.0000	0.0000	0.0000	0.0000	0.0000	0.0000	0.00022
P_Verrucomicrobia	C_Verrucomicrobiae	O_Verrucomicrobiales	F_Rubritaleaceae	G_Luteolibacter	0.0001	0.0003	0.0002	0.0002	0.0000	0.0000	0.0001	0.0015	0.0001	0.0000	0.0001	0.0003	0.0001	0.0002	0.00022
P_Proteobacteria	C_Alphaproteobacteria	O_Rhodobacterales	F_Rhodobacteraceae	G_uncultured	0.0002	0.0002	0.0009	0.0017	0.0000	0.0000	0.0000	0.0000	0.0000	0.0000	0.0000	0.0000	0.0000	0.0000	0.00022
P_Planctomycetes	C_Phycisphaerae	O_Phycisphaerales	F_Phycisphaeraceae	G_SM1A02	0.0011	0.0009	0.0002	0.0000	0.0000	0.0000	0.0000	0.0000	0.0000	0.0001	0.0001	0.0006	0.0000	0.0000	0.00022
Other					0.062	0.070	0.070	0.034	0.032	0.002	0.005	0.030	0.005	0.003	0.008	0.015	0.042	0.008	0.02754

Table S8. Abundance of Bacillus OTUs

OTU	Abundance by stage						
	eggs	larves	female	males	acuatic	adults	all
12404	1325	97	281	0	1422	281	1703
12494	10	11034	1E+05	5258	11044	1E+05	1E+05
12503	127	0	0	1	127	1	128
12745	23	0	2	1	23	3	26
13265	192	0	87	0	192	87	279
15566	1	1	3040	35	2	3075	3077
15962	0	38	342	50	38	392	430
15976	2	14	131	0	16	131	147
16082	0	1	16	1	1	17	18
16231	0	0	4	1	0	5	5
16298	0	0	28	0	0	28	28
16537	0	15	1	0	15	1	16
16692	0	0	3	0	0	3	3
16705	0	0	2	0	0	2	2
17779	0	29	0	0	29	0	29
17803	0	4	2	0	4	2	6
19365	0	0	0	49	0	49	49
20947	18	0	5	0	18	5	23
20969	17	0	0	7	17	7	24
20985	59	2	0	0	61	0	61
21073	40	0	2	0	40	2	42
21084	20	0	0	3	20	3	23
21125	11	1	0	0	12	0	12
21197	379	2	0	0	381	0	381
21226	2	1	2	0	3	2	5
21233	2	0	0	0	2	0	2
21235	2	0	0	0	2	0	2
21307	7	0	0	0	7	0	7
21392	3	0	0	0	3	0	3
21396	5	0	0	0	5	0	5
21574	7	0	0	0	7	0	7
21685	17	0	0	0	17	0	17
21745	125	9	0	0	134	0	134
21761	2	0	0	0	2	0	2
21789	4	1	6	0	5	6	11
22030	2	0	1	0	2	1	3
22092	2	0	0	0	2	0	2
22148	1	7	44	2	8	46	54
22169	39	0	0	7	39	7	46
22441	5	0	0	0	5	0	5
22445	1	0	9	0	1	9	10
22586	3	0	0	0	3	0	3
23129	2	0	0	0	2	0	2
23531	2	0	0	0	2	0	2
23644	2	0	0	0	2	0	2
24792	0	0	31	1	0	32	32
24794	0	1	102	0	1	102	103
24797	0	0	27	1	0	28	28
24858	0	0	3	0	0	3	3
24870	0	0	4	0	0	4	4
24883	0	0	9	0	0	9	9
24901	0	0	2	0	0	2	2
24909	0	0	3	0	0	3	3
24920	0	0	2	0	0	2	2
24947	0	0	2	0	0	2	2

OTU	Abundance by stage						
	eggs	larves	female	males	acuatic	adults	all
24988	0	0	2	0	0	2	2
24989	0	0	2	0	0	2	2
24995	0	1	3	0	1	3	4
25005	0	0	2	0	0	2	2
25055	0	0	6	0	0	6	6
25059	0	0	2	0	0	2	2
25076	0	0	6	7	0	13	13
25081	0	0	3	0	0	3	3
25083	0	1	9	0	1	9	10
25088	0	0	7	0	0	7	7
25121	0	0	3	0	0	3	3
25142	0	0	2	0	0	2	2
25143	0	0	3	0	0	3	3
25205	0	0	18	0	0	18	18
25359	0	0	2	0	0	2	2
25379	0	0	2	0	0	2	2
25389	0	0	4	0	0	4	4
25425	0	0	6	1	0	7	7
25473	0	0	2	0	0	2	2
25489	0	0	1	1	0	2	2
25496	0	0	2	0	0	2	2
25507	0	0	2	0	0	2	2
25536	0	0	2	0	0	2	2
25581	0	0	3	0	0	3	3
25582	0	0	2	0	0	2	2
25586	0	0	3	0	0	3	3
25601	0	0	2	0	0	2	2
25607	0	0	5	1	0	6	6
25672	0	0	2	0	0	2	2
25676	0	0	2	0	0	2	2
25702	0	0	2	0	0	2	2
25703	0	0	2	0	0	2	2
25795	0	0	2	0	0	2	2
25829	0	0	3	0	0	3	3
25852	0	0	2	0	0	2	2
25868	0	0	2	0	0	2	2
25872	0	0	2	0	0	2	2
25905	0	2	4	0	2	4	6
25924	0	0	5	0	0	5	5
25959	0	0	2	0	0	2	2
25980	0	0	2	0	0	2	2
26063	0	0	2	0	0	2	2
26157	0	0	2	0	0	2	2
26198	0	0	2	0	0	2	2
26366	0	0	2	0	0	2	2
26379	0	0	2	0	0	2	2
26384	0	0	2	0	0	2	2
26416	0	0	2	0	0	2	2
26542	0	0	2	0	0	2	2
26551	0	0	2	0	0	2	2
26587	0	0	2	0	0	2	2
26611	0	0	1	1	0	2	2
26656	0	0	2	0	0	2	2
26689	0	0	2	0	0	2	2
26850	0	0	1	1	0	2	2

TS8 Bacillus abundance

OTU	Abundance by stage						
	eggs	larves	female	males	acuatic	adults	all
26864	0	1	1	0	1	1	2
26927	0	0	2	0	0	2	2
26935	0	0	2	0	0	2	2
26943	0	0	2	0	0	2	2
26949	0	4	1	0	4	1	5
26956	0	0	3	0	0	3	3
26971	0	0	2	0	0	2	2
26972	0	0	2	0	0	2	2
26983	0	1	1	0	1	1	2
27117	0	0	2	0	0	2	2
27148	0	1	1	0	1	1	2
27166	0	0	3	1	0	4	4
27235	0	1	1	0	1	1	2
27252	0	0	2	0	0	2	2
27253	0	1	1	0	1	1	2
27264	0	0	2	0	0	2	2
27274	0	0	2	0	0	2	2
27333	0	0	2	0	0	2	2
27411	0	0	2	0	0	2	2
27461	0	0	9	0	0	9	9
27500	0	1	1	0	1	1	2
27517	0	0	2	0	0	2	2
27539	0	0	2	0	0	2	2
27549	0	0	3	0	0	3	3
27619	0	0	6	0	0	6	6
27624	0	0	2	0	0	2	2
27689	0	0	2	0	0	2	2
27711	0	1	2	0	1	2	3
27823	0	1	3	0	1	3	4
27858	0	0	1	1	0	2	2
27874	0	0	1	1	0	2	2
27911	8	0	21	3	8	24	32
28049	0	0	2	0	0	2	2
28139	0	0	2	0	0	2	2
28389	0	0	2	0	0	2	2
28621	0	0	2	0	0	2	2
28649	0	1	1	0	1	1	2
28708	0	0	2	0	0	2	2
28737	0	0	2	0	0	2	2
28903	0	0	2	0	0	2	2
28971	0	1	1	0	1	1	2
29128	0	0	2	0	0	2	2
29286	0	1	1	0	1	1	2
30619	0	3	0	0	3	0	3
30864	0	1	1	0	1	1	2
30866	0	2	0	0	2	0	2
30963	0	2	0	0	2	0	2
31002	0	2	0	0	2	0	2
32705	0	0	0	2	0	2	2
32921	0	0	0	2	0	2	2
33647	4	0	0	0	4	0	4
33731	26	0	0	0	26	0	26
33756	60	2	0	0	62	0	62
33833	6	0	0	0	6	0	6
34060	20	0	0	0	20	0	20
34127	4	0	0	0	4	0	4
34149	14	0	0	0	14	0	14
34215	12	0	0	0	12	0	12

OTU	Abundance by stage						
	eggs	larves	female	males	acuatic	adults	all
34407	2	0	0	0	2	0	2
34434	3	4	0	0	7	0	7
34793	5	0	0	0	5	0	5
35141	10	2	0	0	12	0	12
35406	9	22	3	0	31	3	34
36223	3	0	0	0	3	0	3
36271	8	0	0	0	8	0	8
36293	8	0	0	0	8	0	8
36560	3	8	0	0	11	0	11
36659	2	0	0	0	2	0	2
37813	2	0	0	0	2	0	2
37845	2	0	0	0	2	0	2
39129	2	0	0	0	2	0	2
39208	2	0	0	0	2	0	2
39293	2	0	0	0	2	0	2
39303	4	0	0	0	4	0	4
39445	3	0	0	0	3	0	3
39519	2	0	0	0	2	0	2
39534	3	0	0	0	3	0	3
39711	2	0	0	0	2	0	2
40015	2	0	0	0	2	0	2
40383	4	0	0	0	4	0	4
40506	2	0	0	0	2	0	2
40629	4	0	0	0	4	0	4
41263	2	0	0	0	2	0	2
42309	1	1	0	0	2	0	2
42500	3	0	0	0	3	0	3
43017	3	6	0	0	9	0	9
43417	1	2	0	0	3	0	3
43627	2	0	0	0	2	0	2
45141	2	0	0	0	2	0	2
46178	2	0	0	0	2	0	2
56662	0	0	2	0	0	2	2
69697	0	2	0	0	2	0	2
69904	0	5	0	0	5	0	5
71146	0	2	0	0	2	0	2
1E+05	11	0	0	0	11	0	11
1E+05	53	0	0	0	53	0	53
1E+05	2	0	0	0	2	0	2
1E+05	3	0	0	0	3	0	3
1E+05	2	0	0	0	2	0	2
1E+05	2	0	0	0	2	0	2
2E+05	0	8	0	0	8	0	8
21828	2	0	0	0	2	0	2
21856	4	0	5	1	4	6	10
28647	0	0	2	0	0	2	2

Table S9. Wolbachia sequences summary.

Sample	Amplicon Sequences		cifA		cifB		Recruitmen sequences		
	OTUs	Sequences	sequences	type	sequences	type	wMel	wAlbB	wPip
eggs_s1	1	5					26	25	20
eggs_s2	2	2					43	50	55
larves_s1	5	33					7	9	9
larves_s2	2	3					22	21	20
females_s1	2	3					10	5	4
females_s2	99	33960	93	4	329	16	10166	11952	10435
females_s3	158	49987							
females_s4	2	50							
females_s5	7	109							
males_s1	3	6					9	9	8
males_s2	0	0	2	2	1	1	1471	3591	3102
males_s3	1	1							
males_s4	132	31134							
males_s5	2	3							
